# Changes in Carbon Oxidation State of Metagenomes Along Geochemical Redox Gradients

**DOI:** 10.3389/fmicb.2019.00120

**Published:** 2019-02-11

**Authors:** Jeffrey M. Dick, Miao Yu, Jingqiang Tan, Anhuai Lu

**Affiliations:** ^1^Key Laboratory of Metallogenic Prediction of Nonferrous Metals and Geological Environment Monitoring, Ministry of Education, Central South University, Changsha, China; ^2^School of Geosciences and Info-Physics, Central South University, Changsha, China; ^3^School of Earth and Space Sciences, Peking University, Beijing, China

**Keywords:** geobiochemistry, metagenomics, redox gradient, thermodynamics, chemical composition, environmental shaping, selective degradation, paleoredox

## Abstract

There is widespread interest in how geochemistry affects the genomic makeup of microbial communities, but the possible impacts of oxidation-reduction (redox) conditions on the chemical composition of biomacromolecules remain largely unexplored. Here we document systematic changes in the carbon oxidation state, a metric derived from the chemical formulas of biomacromolecular sequences, using published metagenomic and metatranscriptomic datasets from 18 studies representing different marine and terrestrial environments. We find that the carbon oxidation states of DNA, as well as proteins inferred from coding sequences, follow geochemical redox gradients associated with mixing and cooling of hot spring fluids in Yellowstone National Park (USA) and submarine hydrothermal fluids. Thermodynamic calculations provide independent predictions for the environmental shaping of the gene and protein composition of microbial communities in these systems. On the other hand, the carbon oxidation state of DNA is negatively correlated with oxygen concentration in marine oxygen minimum zones. In this case, a thermodynamic model is not viable, but the low carbon oxidation state of DNA near the ocean surface reflects a low GC content, which can be attributed to genome reduction in organisms adapted to low-nutrient conditions. We also present evidence for a depth-dependent increase of oxidation state at the species level, which might be associated with alteration of DNA through horizontal gene transfer and/or selective degradation of relatively reduced (AT-rich) extracellular DNA by heterotrophic bacteria. Sediments exhibit even more complex behavior, where carbon oxidation state minimizes near the sulfate-methane transition zone and rises again at depth; markedly higher oxidation states are also associated with older freshwater-dominated sediments in the Baltic Sea that are enriched in iron oxides and have low organic carbon. This geobiochemical study of carbon oxidation state reveals a new aspect of environmental information in metagenomic sequences, and provides a reference frame for future studies that may use ancient DNA sequences as a paleoredox indicator.

## 1. Introduction

In the last decade, advances in sequencing technology have produced large metagenomic datasets that can be queried for geobiological information with ever increasing detail. Customarily, studies probe the datasets to find “who is there” and “what are they doing” (Zarraonaindia et al., 2013; Keegan et al., 2016), with the latter question being aided by recent developments in metatranscriptomic analysis. These efforts provide invaluable insight on the identities of organisms, the biogeochemical impacts of their metabolism, and their evolution (Torti et al., 2015). For example, correlations between metagenomic sequences and geochemical conditions have been used to associate community types (Inskeep et al., 2013) or metabolic and evolutionary strategies (Alsop et al., 2014) with environmental variation at different scales. These correlations also imply that systematic changes in the chemical composition of DNA might be present.

As a metric derived from chemical composition, the oxidation state of carbon can be calculated not only for any individual organic molecule (Kroll et al., 2011), but also for complex natural mixtures of organic molecules (Kroll et al., 2015). Previous studies have linked the carbon oxidation state of natural organic matter to the bioenergetics of degradation (LaRowe and Van Cappellen, 2011) and environmental conditions (Boye et al., 2017). Notably, in these studies, correlations of carbon oxidation state with specific environmental redox conditions were shown to emerge as a plausible consequence of thermodynamic constraints. Thermodynamic calculations also offer a route to assess the impacts of redox conditions on microbial metabolism, which is based on oxidation-reduction reactions (e.g., LaRowe and Amend, 2016; Canovas et al., 2017). These and other studies demonstrate strong links between geochemical environments and oxidation states of metabolites and natural organic matter, but it is not known whether geochemistry also shapes the oxidation state of biomacromolecules. Such a finding would imply a new role for geochemical redox gradients in microbial evolution and community structuring.

As with other organic compounds, the carbon oxidation states of biomacromolecules can be calculated from their chemical formulas and therefore from biomolecular sequences (Dick, 2014). Although metabolic reactions are characterized by large changes in oxidation state (in the extreme case of hydrogenotrophic methanogenesis, from +4 for CO_2_ to –4 for CH_4_), the ranges of carbon oxidation state of particular types of biomolecules such as DNA and proteins are much smaller. Nevertheless, systematic patterns at the biomacromolecular level have energetic consequences that can also be quantified through thermodynamics. For instance, it was previously shown that the chemical compositions of metagenomically predicted protein sequences are aligned with the gradients of temperature and redox conditions along the outflow channel of Bison Pool hot spring in Yellowstone National Park (Dick and Shock, 2011, 2013).

Here, we document the changes in carbon oxidation state of metagenomic and metatranscriptomic sequences from datasets representing different types of geochemical redox gradients. In some environments, we find positive correlations between environmental redox gradients and carbon oxidation state of not only DNA, but also RNA and proteins inferred from putative coding sequences. This relationship occurs along mixing paths in hydrothermal systems, in depth profiles in hypersaline lakes, and in the near-surface layers of seafloor sediments and the Guerrero Negro microbial mat. We hypothesize that geochemical redox gradients give rise to thermodynamic constraints that underlie the environmental shaping of the chemical composition of microbial communities.

The proposed thermodynamic constraints are not dominant in all redox gradients, as shown by the increasing carbon oxidation state of metagenomic DNA with depth in the more reducing conditions of marine oxygen minimum zones (OMZs). This pattern is consistent with previously identified low GC content arising from nutrient limitation and genome reduction near the oligotrophic ocean surface (Mende et al., 2017). However, we also observe species-level variability in the carbon oxidation state, which could be a product of horizontal gene transfer or selective degradation of extracellular DNA, if any is present in the samples used for metagenomic analysis. Notably, in the upper 100 m, the carbon oxidation state of proteins increases toward the highly oxic surface waters, which may be a signal of environmental shaping that is not recorded in the chemical composition of DNA. In sediments, more complex patterns in carbon oxidation state emerge, which are probably connected with the onset of more reducing conditions at the sulfate-methane transition zone, but may also reflect different paleoenvironments or a return to relatively aerobic conditions in deeper sediments.

By asking the question, “what are they made of?,” our study reveals widespread systematic behavior of the carbon oxidation states of DNA, RNA, and proteins from metagenomes and metatranscriptomes along geochemical redox gradients. Documenting these trends helps to outline a framework for using the chemical compositions of biomacromolecules as a source of information about the environmental factors that shape microbial communities.

## 2. Methods

### 2.1. Average Oxidation State of Carbon

The theory and applications of carbon oxidation state in organic molecules have been extensively discussed elsewhere (e.g., Kroll et al., 2011; LaRowe and Van Cappellen, 2011; Dick, 2014). The average oxidation state of carbon (*Z*_C_) can be calculated from

(1) $$\begin{array}{r} Z_{\text{C}}=\frac{-h+3n+2o+2s}{c} \end{array}$$

where *c, h, n, o*, and *s* are the coefficients for the corresponding elements in a chemical formula written as C_*c*_H_*h*_N_*n*_O_*o*_S_*s*_ (Dick, 2014).

If the values of *Z* (molecular charge) and *e* (coefficient on phosphorus) are set to zero in the definition of nominal oxidation state of carbon (NOSC) given in equation 4 of LaRowe and Van Cappellen (2011), their equation can be rearranged to write Equation 1. Similarly, with *n* = *s* = 0, Equation 1 yields the formula for carbon oxidation state in common hydrogen- and oxygen-bearing organic molecules (${\bar{\text{OS}}}_{\text{C}}$; Kroll et al., 2011). Ionization by gain or loss of protons, or dehydration reactions associated with the polymerization of amino acids to form proteins, do not alter *Z*_C_ (Dick, 2014). The addition of a phosphate group to either the 3' or 5' end of a deoxyribose or ribose molecule is likewise a dehydration reaction, and the resulting sugar-phosphate backbone involves no C–P bonds. Therefore, accounting for the charged phosphate groups is not necessary to calculate the *Z*_C_ of a strand of DNA or RNA, which is simply equal to that in the constituent nucleosides.

### 2.2. Sequence Processing

Nucleic acid FASTA files of unassembled reads were downloaded from the NCBI Sequence Read Archive (SRA) or the MG-RAST server (Meyer et al., 2008). Following previous recommendations (Brazelton and Baross, 2009), we preferred to use unassembled sequences, because assembly loses the frequency information needed for comparative metagenomics (Meyer et al., 2008). However, for a few datasets that are important representatives of their environments (Bison Pool, Guerrero Negro, Shimokita Peninsula, and Yellowstone Park), only contigs rather than individual reads could be found in public databases; these were downloaded from NCBI GenBank or IMG/MER (Chen et al., 2017). The reads were processed using scripts based on an adaptation of the MG-RAST pipeline for FASTA files of metagenomic shotgun sequences (Meyer et al., 2008; Wilke et al., 2017) that terminates after the RNA and protein gene-calling steps; that is, no taxonomic or functional annotation was performed at this stage. The scripts were downloaded from GitHub^1^ and utilized via a workflow that was implemented in R (R Core Team, 2018). This file (named ARAST.R for “Abbreviated RAST”) and all other code and data files required to reproduce the calculations in this paper have been deposited in the Zenodo repository (Dick et al., 2018).

Adapter trimming was carried out using the autoskewer.py script, which utilizes the Skewer program (Jiang et al., 2014). Length filtering (removal of sequences with length outside of two standard deviations of the mean, or with more than 5 ambiguous bases) was performed using the filter_sequences command with parameters taken from the mgrast_preprocess.pl script. Dereplication was carried out using the dereplication.py script. rRNA gene calling was performed using the arast_sortme_rna.pl script, which depends on the SortMeRNA program (Kopylova et al., 2012). This script was modified from MG-RAST's mgrast_sortme_rna.pl to save both rRNA and non-rRNA sequences. The sequences remaining after the dereplication step and the non-RNA sequences identified by the rRNA gene calling step were used for calculation of *Z*_C_ of DNA in metagenomes and metatranscriptomes, respectively. These sequences were base-paired to obtain the nucleobase composition of double-stranded DNA, which was used to compute *Z*_C_. Double-stranded DNA (dsDNA) was used in this calculation to more accurately represent the composition of genomic DNA, since metagenomic reads represent the sequences of single strands of fragmented DNA, and there may be significant GC skew between the leading and lagging strands in bacterial DNA (Lobry, 1996).

Most datasets considered in this study are composed of unassembled reads. For these datasets, following MG-RAST, dereplication was used to remove artificial duplicate reads (ADR) from the datasets (Keegan et al., 2016). Duplicated reads can be especially abundant when extra PCR cycles are used for amplification, such as in a metagenome study of the Baltic Sea sediment (Marshall et al., 2018). Although read coverage of assemblies is an important consideration for comparing relative abundances of genes or organisms, the main focus of this study is on whole metagenomes. Therefore, base frequencies and carbon oxidation state were calculated for all reads remaining after dereplication without any weighting.

Protein gene calling was performed using the parallel_FragGeneScan.py script, which depends on the FragGeneScan program (Rho et al., 2010). In contrast to MG-RAST, which detects overlap between putative protein-coding genes and rRNA genes (Wilke et al., 2017), our workflow just uses the non-rRNA sequences, as identified by SortMeRNA, for the protein gene calling step. The nucleic acid and amino acid sequence files produced by FragGeneScan were used for calculation of *Z*_C_ of putative mRNA and proteins in both metagenomes and metatranscriptomes. The subset of negative-sense DNA sequences identified by FragGeneScan was complemented, then the nucleobase composition of mRNA corresponding to the entire (now positive-sense) set of coding DNA sequences was used to calculate *Z*_C_ of mRNA.

Intra-sample variation was calculated as the standard deviation of the carbon oxidation state for random subsamples of sequences in each sample. Subsamples were generated having, on average, a total of 50,000 bases or amino acids. Subsampling was performed 100 times; then, *Z*_C_ was computed for each of the subsamples, and the mean and standard deviation of *Z*_C_ were used to draw the lines and error bars on the plots. Note that the subsamples generally represent only a small fraction of the total metagenomic data; increasing the subsample size yields smaller error bars, but has a secondary effect on the computed mean values. The Zenodo data deposition (Dick et al., 2018) contains the nucleobase and amino acid compositions computed from the subsampling.

For sequence files of contigs downloaded from GenBank or IMG, the steps up to and including rRNA gene calling were skipped, and only the calculation of dsDNA composition and protein gene calling were performed. Sequence processing statistics and accession numbers for all datasets used in this study are provided in Table S1. Due to limited computational resources, we generally used partial FASTA files (up to 150 MB uncompressed size) downloaded from SRA. The total number of available reads and the number of used reads for each dataset is given in Table S1. For estimating the chemical composition of DNA and proteins in the whole metagenome, partial files are sufficient. However, complete SRA files were used for taxonomic analysis (see below), and also for the calculation of *Z*_C_ for whole metagenomes presented in **Figure 5**. That figure shows similar results to the calculations based on partial files.

### 2.3. Thermodynamic Calculations

The overall synthesis of different protein or DNA sequences from inorganic constituents can be represented by writing formation reactions from a set of basis species, then comparing the chemical affinities (opposite of overall Gibbs energy, Δ*G*) of these reactions to assess the relative stabilities of the molecules in a given environment as defined by the temperature and activities of the basis species. Although a group additivity model for proteins including provision for variable ionization of side chains is available (Dick et al., 2006), an analogous model is not available for DNA, and a simplified additive estimate of thermodynamic properties taking account of the frequencies of monomers was used for both proteins and DNA in this study. Frequencies of amino acids in predicted proteins and base pairs in double-stranded DNA were retrieved from the processed metagenomic and metatranscriptomic data and were combined with standard Gibbs energies of amino acids (Dick et al., 2006) or +2 charged nucleotide monophosphates (LaRowe and Helgeson, 2006) at 25 °C to give an average per-monomer chemical formula and standard Gibbs energy of the biomacromolecules in each sample. Average per-monomer reactions were written for the formation of DNA and proteins from these basis species (with constant logarithms of chemical activity): H_2_O (0), $\text{HC}{\text{O}}_{3}^{-}$ (–3), ${\text{H}}_{2}\text{P}{\text{O}}_{4}^{-}$ (–5), $\text{N}{\text{H}}_{4}^{+}$ (-7), HS^−^ (–9), H^+^ (–7, i.e., pH = 7), and *e*^−^ (represented by Eh, which is used as a variable in the plots). Chemical affinities of the reactions were calculated as a function of Eh at 25 °C and 1 bar using the CHNOSZ software package (Dick, 2008).

A hypothetical protein consisting of 50 alanines (C_3_H_7_NO_2_; *Z*_C_ = 0) and 50 glycines (C_2_H_5_NO_2_; *Z*_C_ = 1) would be represented in the model by a chemical formula that is the average of these amino acids (C_2.5_H_6_NO_2_), which has a *Z*_C_ of 0.4. If the glycines were replaced by leucine (C_6_H_13_NO_2_; *Z*_C_ = −1), the per-monomer formula of the protein would be C_4.5_H_10_NO_2_, which is considerably more reduced (*Z*_C_ = −0.67) and whose synthesis would therefore be predicted to be energetically favored relative to the first protein by a shift toward a more reducing environment. Although this per-monomer model does not account for the loss of H_2_O upon polymerization of amino acids or nucleotides, a single H_2_O is lost for each monomer, and therefore cancels out in the calculation of relative affinities; furthermore, dehydration reactions do not affect the *Z*_C_ of the molecules.

### 2.4. Taxonomic Classification

Taxonomic classification was performed using Kraken (Wood and Salzberg, 2014) with the “dustmasked” 8 GB MiniKraken database^2^. In order to obtain sufficient numbers of reads to represent the chemical compositions of DNA in individual species, complete DNA sequence FASTA files were obtained using NCBI's SRA Toolkit (version 2.9.0)^3^. Source FASTA files were processed by trimming, filtering, and dereplication as described above, then analyzed with Kraken. The kraken-report command was used to produce summaries of taxonomic classifications, which were scanned to identify taxa at the species or subspecies level making up at least 1% of the classified sequences in any sample. The summaries for these species are provided in the Zenodo data deposition (Dick et al., 2018). Three to six species were selected for each dataset, with preference given to species that are present and relatively abundant in multiple datasets. The reads classified to each species were subsampled 100 times with a sample size yielding 10,000 bases on average. Because of the limited numbers of reads for individual species, the subsample size must be smaller than that used for the first part of the study, leading to a higher standard deviation of the computed *Z*_C_ values. The accession numbers used, taxonomic IDs of species, and numbers of reads classified to each species are given in Table S2. For this analysis we used a recent metagenome for the HOT ALOHA station (Mende et al., 2017) that is larger than the one used for the first set of calculations displayed in Figures S1, S2 (Shi et al., 2011).

Accurate classification of shotgun metagenomic sequences at the species level can be problematic, but we believe that the methods used here provide a reasonable estimate of the composition of selected species. First, the reads used for taxonomic classification are not extremely short. The average length of classified reads was about 215 bp for Ginger Castle and Shrimp Gulley 2 in the Diffuse Vents datasets, 300 bp for other datasets in the Diffuse Vents, 460–500 bp for Menez Gwen, 350–460 for ETNP_OMZ, 260–280 for ETSP_OMZ, and 300 for HOT ALOHA (Table S2). Read lengths of at least around 250 bp are needed to improve the sensitivity of many classification methods, but increasing lengths do not have a large impact on their precision (Peabody et al., 2015).

Second, Kraken is notable for using an exact *k*-mer matching algorithm that results in very high precision (Wood and Salzberg, 2014). Compared to alignment-based methods, Kraken was shown to have higher precision at the genus level for Illumina HiSeq metagenomic data with average read lengths as short as 92 bp (Wood and Salzberg, 2014). While the precision can be expected to drop by a few percent in species- compared to genus-level classification, the drop in sensitivity is considerably larger, as shown for classification of ribosomal RNA subunits using the MiniKraken database (Martínez-Porchas et al., 2016). The main drawback with our method is that the sensitivity is quite low, as manifested by the low classification rate in our analysis (median 2.2%; see Table S2). This low classification rate, combined with our requirement for sufficient total number of base pairs in all reads classified to a single species (20,000 bp, which is double the subsample size indicated above) to compute the average carbon oxidation state, greatly limits the number of species we can include for comparative analysis. Although this method suffers from a low sensitivity (high numbers of false negatives), it has a high precision (low numbers of false positives) that should give a low error in the calculation of representative chemical compositions for metagenomic DNA of individual species.

## 3. Results and Discussion

### 3.1. Environmental Context

Metagenomic and metatranscriptomic data available in public databases were selected to represent different types of geochemical redox gradients. Sediments, hydrothermal systems, microbial mats, and stratified water bodies provide some of the most well recognized examples of redox gradients, so we have focused on these environments. Soils are another example, but they have very complex communities, and we chose not to include them in this study.

An important criterion for selection was the availability of corresponding measurements of oxygen, hydrogen, sulfate, methane, or other redox-sensitive species. The Appendix describes the sources of sequencing data and the general redox characteristics of the environments. In summary, the datasets represent ocean oxygen minimum zones in the Eastern Tropical North Pacific (ETNP) (EN; Ganesh et al., 2015; Glass et al., 2015) and Eastern Tropical South Pacific (ETSP) (ES; Stewart et al., 2012), relatively oxygenated ocean water at Hawaii Ocean Time-Series (HOT) station ALOHA (HA; Shi et al., 2011), mixing of seawater and hydrothermal fluid from diffuse vents on the Mid-Cayman Rise and Juan de Fuca Ridge (DV; Reveillaud et al., 2016; Fortunato et al., 2018) and Menez Gwen on the Mid-Atlantic Ridge (MZ; Meier et al., 2016), seafloor sediments of the Baltic Sea (BS; Thureborn et al., 2016; Zinke et al., 2017; Marshall et al., 2018) and offshore Shimokita Peninsula in Japan (SP; Kawai et al., 2014), rock-derived fluids in serpentinite springs (SS; Brazelton et al., 2012), the Shin-Yan-Ny-Hu (SYNH) terrestrial mud volcano in southwestern Taiwan (MV; Cheng et al., 2012), stratified hypersaline environments in Mono Lake, California (ML; Edwardson and Hollibaugh, 2017) and Organic Lake, Vestfold Hills, Antarctica (OL; Yau et al., 2013), hot springs in Yellowstone National Park including the outflow channel of Bison Pool (BP; Havig et al., 2011; Swingley et al., 2012) and different community types from multiple hot springs (YP; Inskeep et al., 2013), and the microbial mat in Guerrero Negro, Baja California Sur, Mexico (GN; Kunin et al., 2008).

The environmental conditions generally become more reducing deeper into water, sediments, or microbial mats (Nealson and Stahl, 1997; see also the references for individual datasets in the Appendix). In submarine hydrothermal systems, the conditions are more reducing at lower seawater mixing ratios, which occur closer to the vents. Near-millimolar H_2_ is also an indicator of more reducing conditions at the Ginger Castle and Shrimp Gulley #2 diffuse vent sites on the Mid-Cayman rise (Reeves et al., 2014) compared to diffuse vents on the Axial Seamount on the Juan de Fuca Ridge, where near-micromolar concentrations of H_2_ were reported (Fortunato et al., 2018). At Bison Pool in Yellowstone National Park, different proxies for redox conditions (dissolved oxygen, sulfate/sulfide ratios and electrode measurements of oxidation-reduction potential (ORP)) indicate a redox gradient that is more oxidizing going away from the source pool (Dick and Shock, 2011). In the comparison of multiple hot springs in Yellowstone National Park, the presence of sulfide and/or elemental S is taken as a proxy for more reducing conditions (Inskeep et al., 2013). In this paper, we use “oxidizing” and “reducing” to refer to environmental oxidation-reduction conditions, and “oxidized” and “reduced” to indicate the relative carbon oxidation states of biomolecules.

Because geochemical and metagenomic analyses depend on different physical samples, there are limitations in comparing the two. However, the errors introduced to the comparisons should be relatively small in datasets where samples are separated by large distances, such as depth transects in oceans. At sampling scales of centimeters or smaller (e.g., Menez Gwen, Mud Volcano, Guerrero Negro), there is likely to be a larger uncertainty associated with comparisons of geochemistry and metagenomic data from different samples. Nevertheless, we anticipate that this type of uncertainty is secondary to our main observation of changes carbon oxidation state that span multiple samples along geochemical gradients. For instance, at Menez Gwen, where the most distal sample was taken 40 cm from the vent, the oxygen, hydrogen, methane, and H_2_S concentrations reported by Meier et al. (2016) are estimates derived from a mixing model, as *in-situ* measurements are not available for all samples. Our observation of a correlation with biomolecular carbon oxidation state does not depend on the absolute correctness of these values, only on the redox gradient, which clearly becomes more oxidizing with greater seawater mixing. However, we are aware that the thermodynamic model described here uses a single range of Eh that is probably not realistic for all environments, and should be adjusted in future refinements of the model.

### 3.2. General Characteristics of Carbon Oxidation State of DNA and RNA

**Figure 1:**
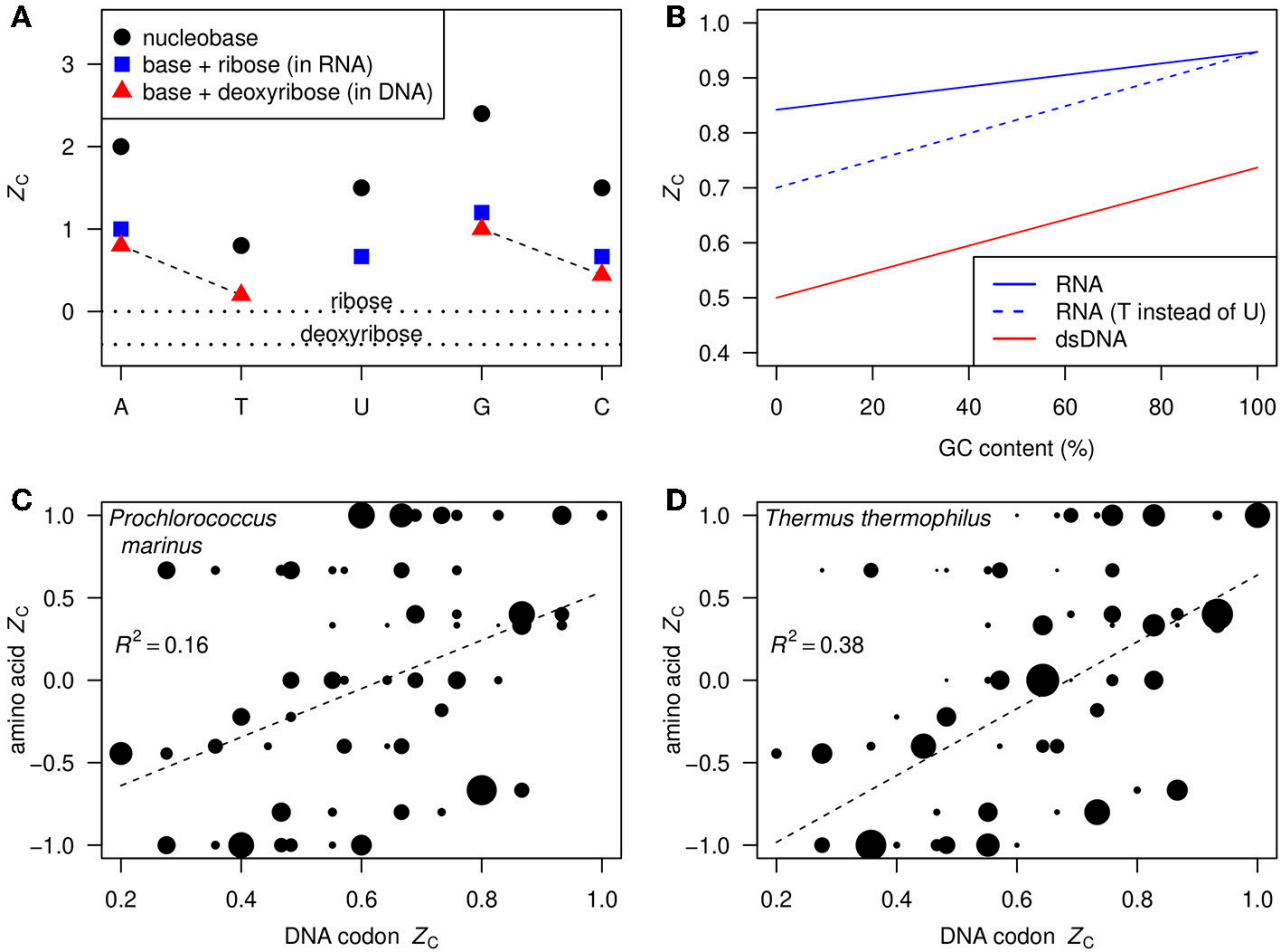
**(A)** *Z*_C_ (Equation 1) of nucleobases (black circles), ribose and deoxyribose (horizontal dotted lines), and nucleosides in RNA and DNA (blue squares and red triangles). The dashed lines indicate the base pairs in DNA. **(B)** *Z*_C_ as a function of GC content in double-stranded DNA (red line) and single-stranded RNA assuming equal abundances of G and C and of A and U (blue line). As a thought experiment, the dashed blue line represents hypothetical single-stranded RNA where T takes the place of U; the constant displacement from the red line represents the difference between DNA and RNA that is due only to the substitution of deoxyribose by ribose. **(C,D)** Scatterplots of *Z*_C_ of DNA codons (not double-stranded) and corresponding amino acids. Areas of points are proportional to the frequencies of the codons in the indicated organisms, and regression lines are plotted using the frequencies as weighting factors.

Figure 1A shows the *Z*_C_, calculated using Equation 1, of each of the nucleobases and those of the corresponding nucleosides in RNA and DNA. The *Z*_C_ of the nucleosides is intermediate between the relatively high *Z*_C_ of the nucleobases, ranging from 0.8 for thymine to 2.4 for guanine, and the relatively low *Z*_C_ of ribose and deoxyribose, which are 0 and –0.4, respectively. Also indicated in Figure 1A are the A–T and G–C base pairs in DNA; the G–C pair has a higher carbon oxidation state (0.74) than the A–T pair (0.50).

In double-stranded DNA, there is a linear relation between *Z*_C_ and GC content (percentage of bases that are either G or C), as shown by the red line in Figure 1B. GC content in whole genomes ranges from approximately 25–75% (Wu et al., 2012). Given this range and the relation shown in Figure 1B, we predict that *Z*_C_ values for most bulk DNA are about 0.56–0.68. Substituting ribose for deoxyribose contributes to increase the *Z*_C_ of RNA by 0.2 over that of DNA, as indicated by the dashed blue line in Figure 1B. Likewise, uracil in RNA is more oxidized than thymine in DNA. This yields another positive contribution to *Z*_C_ of RNA that is greater at low GC content (solid blue line in Figure 1B).

In contrast to double-stranded DNA, the *Z*_C_ of single-stranded DNA and RNA depends on the relative abundances of all bases, not only GC content. The total range is apparent in Figures 1C,D, showing the *Z*_C_ of the 61 amino acid-coding DNA codons and the corresponding amino acids. The point sizes in these plots reflect the codon usage frequencies in *Prochlorococcus marinus* str. AS9601 and *Thermus thermophilus* HB8 using data from the Codon Usage Database (Nakamura et al., 2000)^4^. The codon usage differs considerably between these mesophilic marine and thermophilic terrestrial organisms. However, in both cases the *Z*_C_ of amino acids is moderately correlated with that of the DNA codons, so we expect to find an overall correlation between *Z*_C_ of metagenomic DNA and the proteins inferred from putative coding sequences.

### 3.3. Carbon Oxidation State Along Geochemical Redox Gradients

We calculated the average oxidation state of carbon (*Z*_C_) in biomolecular sequences obtained from different redox gradients. Chemical formulas for sequences of double-stranded DNA (computed by base-pairing the metagenomic sequences) and inferred sequences of messenger RNA and proteins were used to calculate *Z*_C_ (see section Methods for details).

**Figure 2:**
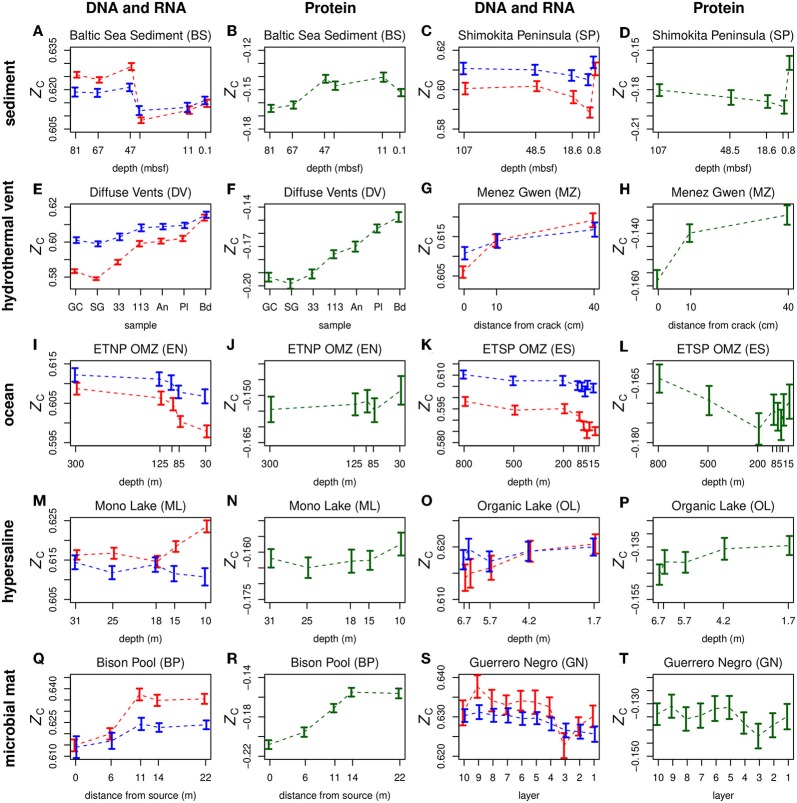
Carbon oxidation state (*Z*_C_) of double-stranded DNA (red symbols), messenger RNA of predicted coding sequences (blue symbols), and proteins (green symbols) along geochemical redox gradients. Separate plots for nucleic acids and proteins are provided for two datasets each for sediment **(A-D)**, hydrothermal vent **(E-H)**, ocean **(I-L)**, hypersaline **(M-P)**, and microbial mat **(Q-T)** environments. In order to plot both DNA and RNA on the same diagram, a constant of 0.28 was subtracted from *Z*_C_ of RNA. The horizontal axis in each plot is ordered so that relatively oxidizing conditions are toward the right-hand side. This figure includes selected metagenomic datasets representing different types of environments, as indicated by the row titles. The Mono Lake dataset is a metatranscriptome. Plots for all datasets considered in this study are in Figure S1 (DNA and RNA) and Figure S2 (proteins). Abbreviations for sample names are given in the Appendix.

Plots of *Z*_C_ of DNA, RNA, and proteins along geochemical redox gradients in ten representative datasets for different environments are shown in Figure 2. Plots for all 18 datasets considered in this study are provided in Figure S1 for DNA and RNA and Figure S2 for proteins. The dashed lines connect the mean values for different samples in a dataset. Because RNA inherently has a higher *Z*_C_ than DNA (see Figure 1B), an offset of −0.28 was applied to the *Z*_C_ of RNA in order to show both DNA and RNA (in red and blue, respectively) on the plots.

In Figure 2 and Figures S1, S2, the horizontal axes are ordered so that samples with more oxidizing conditions are positioned toward the right-hand side. This arrangement allows a quick visual comparison of carbon oxidation state with the overall redox gradient in each dataset. Most datasets exhibit either a positive or negative overall correlation, while others, such the Guerrero Negro microbial mat and the sediments offshore Shimokita Peninsula, exhibit a more complex behavior. The plots also reveal generally parallel trends between the carbon oxidation states of DNA and RNA and, for some datasets, proteins.

The relatively oxidizing surface zones of many environments often exhibit significant increases in biomolecular carbon oxidation state compared to regions just below the surface. Examples are provided by both DNA and inferred proteins from the Guerrero Negro microbial mat (Figures 2S,T) and proteins from the ETNP and ETSP oxygen minimum zones (OMZ) (Figures 2J,L). Moreover, an Antarctic hypersaline lake (Organic Lake) and the SYNH Mud Volcano in Taiwan both display increases of *Z*_C_ of DNA and proteins going from the deepest samples to the surface (Figures 2O,P; Figures S1, S2). The seawater background of the Diffuse Vents and the distal sample at Menez Gwen are other relatively oxidizing environments that yield DNA and proteins with relatively high *Z*_C_ (Figures 2E–H). For stratified systems considered in this study, the only metagenomic dataset where the *Z*_C_ of proteins does not increase at the surface is the Baltic Sea sediment (Figure 2B), but an increase can be detected in the metatranscriptomic data (Figure S2).

### 3.4. Comparison of Metagenomic DNA and Putative Proteins

**Figure 3:**
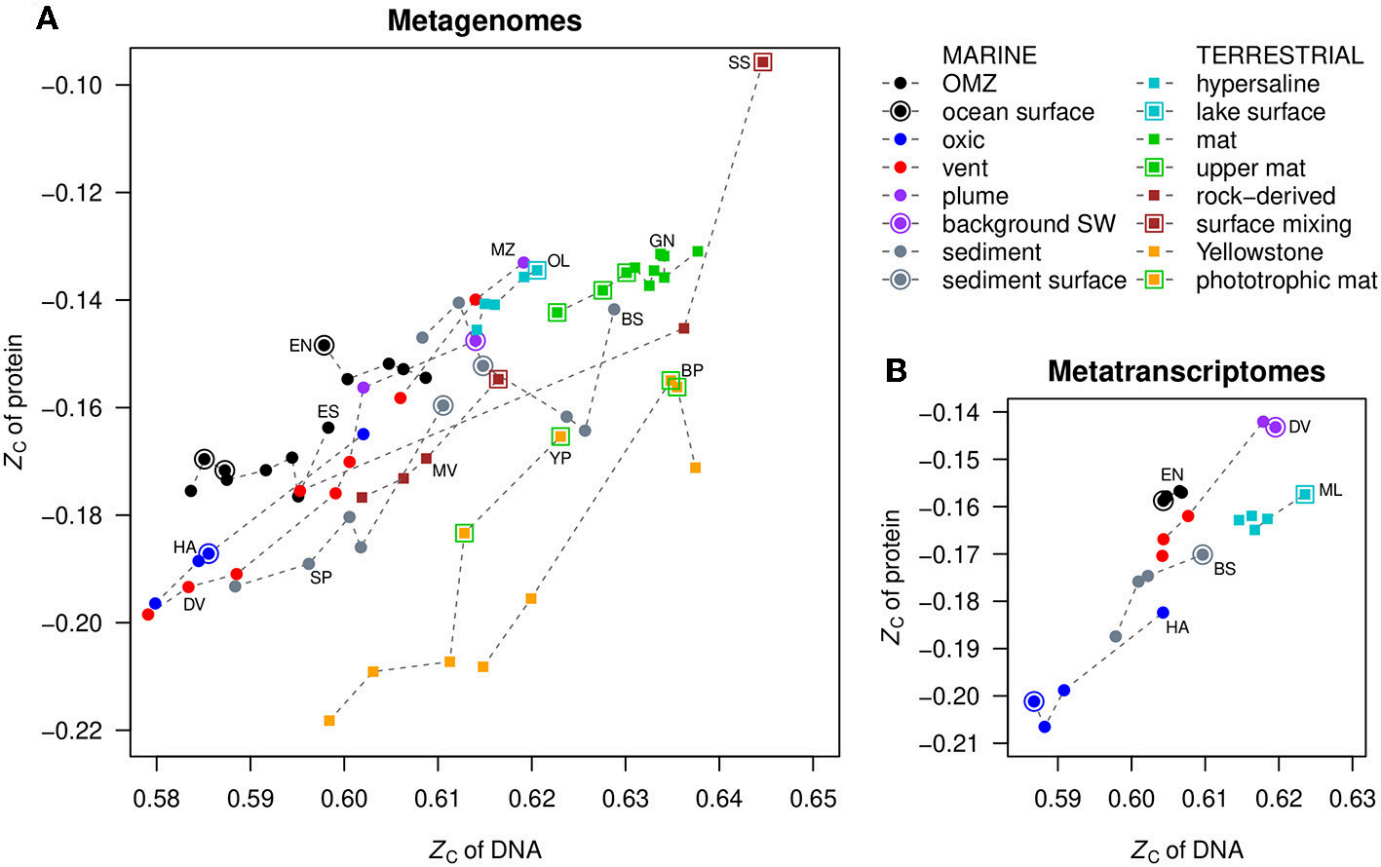
Comparison of mean values of carbon oxidation state (*Z*_C_) of DNA and proteins in **(A)** metagenomes and **(B)** metatranscriptomes. Dashed lines connect points in the same dataset, ordered by *Z*_C_ of DNA; this does not necessarily correspond to the spatial order of samples. OMZ, oxygen minimum zone; SW, seawater.

The mean values of carbon oxidation state of metagenomic DNA and inferred proteins are compared with each other in Figure 3A. The protein sequences were obtained from FragGeneScan (see section Methods) and reflect putative proteins coded by the metagenome, not necessarily those that are actually expressed or present in the communities. Notably, for datasets where *Z*_C_ of DNA is positively correlated with the redox gradient (that is, increases toward more oxidizing conditions; see Figure 2 and Figure S1), the *Z*_C_ of proteins also parallels that of DNA. This is most apparent for Diffuse Vents, Menez Gwen, SYNH Mud Volcano, Organic Lake, Serpentinite Springs, and Yellowstone Park. The samples for ocean surface and seawater endmembers, as well as near-surface samples in terrestrial environments, are indicated by outlined symbols in Figure 3, emphasizing their locally higher *Z*_C_.

Phototrophic mats in Yellowstone National Park are highlighted by green outlines in Figure 3. This is another relatively oxidizing environment that is distinguished from the chemotrophic communities that inhabit the hotter, more reducing waters near the sources of hot springs. The metagenomic data for Bison Pool (Havig et al., 2011; Swingley et al., 2012) reveal the transitions of communities along the outflow channel of a single hot spring, while the Yellowstone Park dataset (YNP metagenome project; Inskeep et al., 2013) was obtained from different hot springs. Despite the differences in study design, both datasets show the same overall trends toward more oxidized DNA, RNA, and proteins in the cooler and more oxidizing phototrophic zones (Figures S1, S2).

In contrast to the positive correlations described above, in the oxygen minimum zones of oceans (ETNP and ETSP) and the deeper layers of the Guerrero Negro microbial mat, negative correlations are apparent: the *Z*_C_ of metagenomic DNA increases despite the occurrence of more reducing conditions with depth (Figures 2I,K,S). The *Z*_C_ of proteins in these datasets shows little correlation with that of DNA, but the *Z*_C_ of proteins increases at the surface compared to just below the surface (Figures 2J,L,T, 3A). The dataset for HOT ALOHA, where there is detectable oxygen at all depths (Shi et al., 2011), exhibits a higher *Z*_C_ of DNA at lower oxygen concentrations, similar to the OMZs, but in contrast has a positive correlation between *Z*_C_ of DNA and proteins (Figure 3A).

### 3.5. Carbon Oxidation State of Metatranscriptomes

The trends described above are reflected to a large extent in the metatranscriptomic datasets (Mono Lake in Figures 2M,N, and plots labeled “MT” in Figures S1, S2). The *Z*_C_ of the expressed genes detected in metatranscriptomes increases toward the sediment surface in the Baltic Sea sediment and the water surface in Mono Lake. Relatively high carbon oxidation states of metatranscriptomic cDNA are also apparent for background seawater compared to hydrothermal fluids in the Diffuse Vents dataset. Although the changes of *Z*_C_ of DNA and RNA along redox gradients are to some extent correlated, some differences between them are apparent in Figure 2 and Figure S1. For metatranscriptomic datasets in particular (Figure S1), the changes in *Z*_C_ of RNA are relatively flat; this may reflect physiological requirements that limit the range of chemical composition of messenger RNA more than genomic DNA.

Notably, each of the metatranscriptomic datasets considered here exhibits a positive overall correlation between *Z*_C_ of DNA and proteins (Figure 3B). Such a trend might be expected based on the general correlation between carbon oxidation state of codons and amino acids (see Figures 1C,D). However, the tightly coupled variation of *Z*_C_ of transcribed DNA and proteins in many metatranscriptomic datasets suggests the possibility of external forces that may shape the chemical compositions of both types of biomolecules.

### 3.6. Thermodynamic Aspects of Correlations Between DNA, Proteins, and Environments

In terms of ATP requirements, the biosynthesis of G and C is more demanding than A and T (Rocha and Danchin, 2002), but for cells that are in close contact with the environment, the energetics of synthesis reactions depend on environmental factors (LaRowe and Amend, 2016). For instance, the overall Gibbs energies (Δ*G*) of synthesis of different nucleobases, amino acids, and other biomolecules are sensitive to fluid composition along a seawater-hydrothermal fluid mixing path (Shock and Canovas, 2010). Here we use thermodynamic calculations to characterize the potential for environmental constraints on the carbon oxidation states of DNA and proteins.

**Figure 4:**
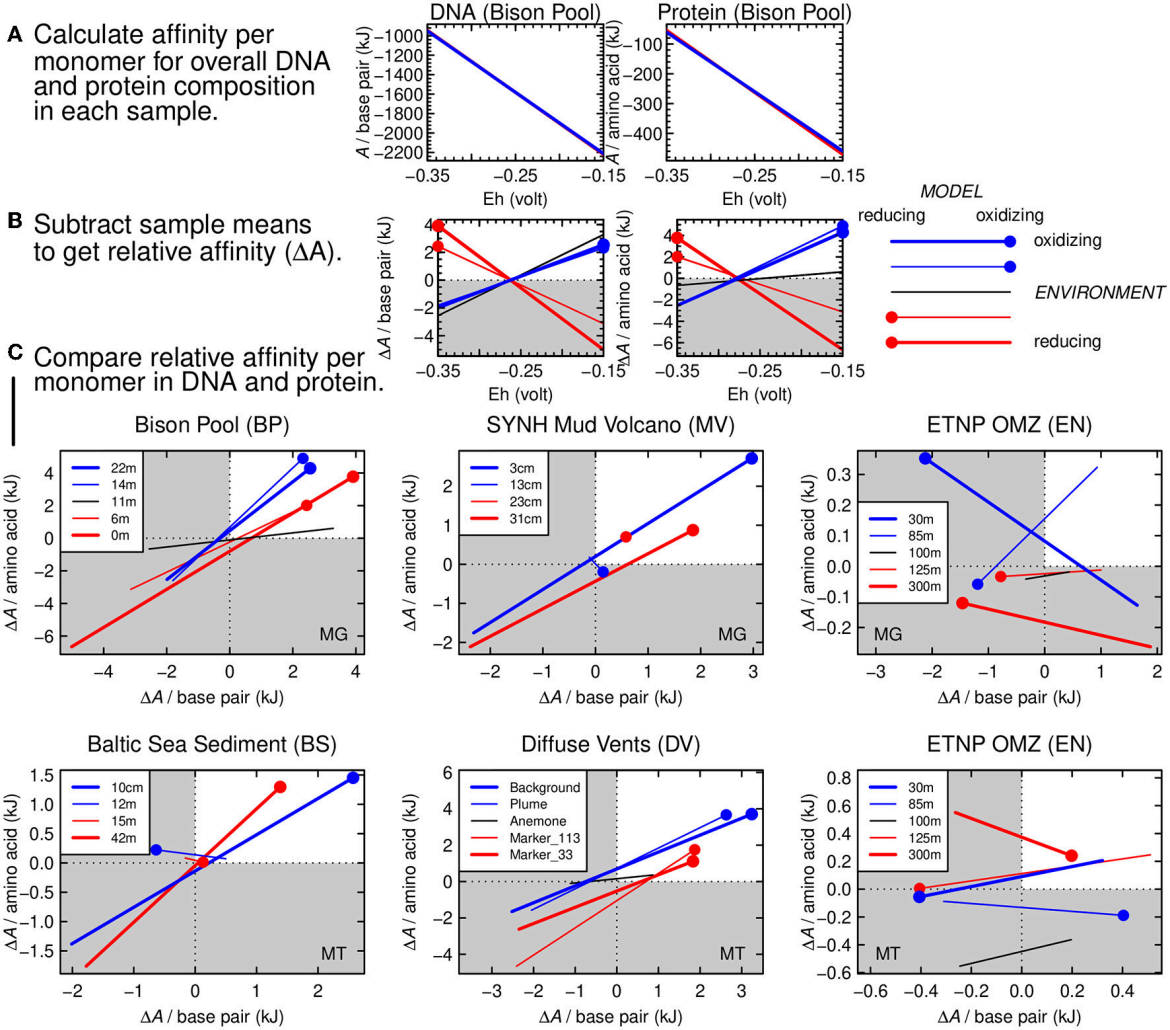
Thermodynamic calculations for relative potential for synthesis of DNA and proteins as a function of environmental oxidation-reduction conditions. **(A)** Thermodynamic potential (chemical affinity) calculated for formation reactions of average monomer compositions of DNA (nucleotide monophosphate base pairs) and proteins (amino acids) at Bison Pool. The *x*-axis shows the variation of Eh in the calculations. Lines for all 5 samples are present in each plot (red: relatively reducing environment; blue: relatively oxidizing environment) but are nearly indistinguishable from each other. **(B)** Relative chemical affinity of formation per monomer of DNA and proteins in each sample calculated by subtracting the mean value for all samples from the individual sample values. Red circles on the red lines indicate reducing model conditions for the relatively reducing environments; blue circles and lines correspond to oxidizing conditions. **(C)** Cross-plots of relative affinity of formation of monomers in DNA and proteins for different metagenomic and metatranscriptomic datasets, starting with Bison Pool. In quadrant I (white), the relative affinities of formation of DNA and proteins are both positive, indicating a viable thermodynamic model.

The chemical affinities (i.e., the opposite of overall Gibbs energy; *A* = −Δ*G*) of reactions representing the synthesis of biomolecules from inorganic precursors are shown in Figure 4A for the five sampling sites in the Bison Pool dataset. These sites follow the gradient from relatively hot, reducing conditions at the source pool to cool, oxidizing conditions farther along the outflow channel. As indicated by higher values of *A* at lower values of redox potential (Eh), reactions to synthesize the average per-monomer DNA and protein composition at all sites become more favorable at more reducing conditions. This result is compatible with previous calculations of the energetics of biomolecular synthesis (e.g., LaRowe and Amend, 2016). The different lines in Figure 4A, representing each of the five sampling sites at Bison Pool, are nearly indistinguishable from each other because of the large dependence of the energetics of oxidation-reduction reactions on the redox variable (Eh) and the use of average per-monomer compositions of both DNA and proteins. These average compositions of biomacromolecules span a smaller compositional range than the individual monomers that make up the sequences. This averaging step is a way to normalize the chemical formulas; without it, energetic differences related to compositional variation would be obscured by the different sizes of the biomacromolecules (Dick, 2008).

The plot in Figure 4A would look much the same for any other dataset. However, there are small yet important differences in the energetics of the reactions that are not easily seen in Figure 4A. To visualize these differences, we calculated the mean value of the chemical affinity for all samples at each point along the Eh scale. This sample mean, or virtual baseline, was subtracted from the chemical affinity of the samples themselves (Figure 4A) in order to produce the relative affinities shown in Figure 4B. The blue lines in this figure stand for the two most oxidizing samples (farthest from the source of Bison Pool), and the red lines stand for the two most reducing samples (closer to the source). Thicker red and blue lines are used to indicate the most extremely located samples. The black line represents the sample from the transition zone at Bison Pool, known as the “photosynthetic fringe” (Havig et al., 2011; Swingley et al., 2012). The red and blue dots in Figure 4B are placed on the same colored lines at the corresponding (reducing and oxidizing) limits of the Eh scale on the plot. It is apparent that DNA and proteins from the samples closer to the source of the hot spring have a positive relative affinity at reducing conditions (low Eh), and those from the cooler, more oxidizing parts of the hot spring have a positive relative affinity at more oxidizing conditions (high Eh).

For rapidly assessing many datasets it is more convenient to visualize the results for DNA and proteins in a single plot. This can be done by plotting only the value of the *y*-axis variables in Figure 4B (relative chemical affinity per base pair or amino acid) as the two axes of the plots in Figure 4C. The points at the ends of the lines in Figure 4C have the same meaning as those in Figure 4B, and provide an anchor that indicates which end of the lines corresponds to the upper or lower limit of the Eh scale. For Bison Pool, these dotted ends all lie in the first quadrant, which indicates that the relative reaction energies are aligned with the environmental conditions.

There are only four samples for the Mud Volcano, so no black line is present for this dataset in Figure 4C. The two samples at intermediate depths (13 and 23 cm) are represented by thinner red and blue lines that are relatively short; the red one is very short and only the dot is visible. This signifies that the energetics of the overall synthesis of DNA and protein in these samples, relative to the deepest and shallowest samples, are less sensitive to changes of redox potential.

In Figure 4C, the first (upper-right) quadrant represents a positive relative affinity. A model that “hangs together” is indicated when the dotted ends of the lines, corresponding to either oxidizing or reducing conditions, fall in the first quadrant. The metagenomes of Bison Pool and SYNH Mud Volcano and the metatranscriptomes of Baltic Sea sediment and the Diffuse Vents all have this pattern. Therefore, a plausible geobiochemical hypothesis is that environmental shaping of the carbon oxidation state of metagenomic DNA and proteins arises from thermodynamic constraints associated with geochemical redox gradients. Because the thermodynamic model includes both DNA and proteins, this hypothesis can account for the coupled changes in carbon oxidation states of both types of biomacromolecules in these settings (Figure 3).

As a counterexample, the carbon oxidation states of DNA and proteins in the metagenomes and metatranscriptomes of the ETNP oxygen minimum zone are not positively correlated with the environmental redox conditions (Figures 2I,J; Figures S1, S2). Consequently, the relative chemical affinities for the synthesis of DNA and proteins plot as a scattered arrangement of the lines in Figure 4C. In this case, the thermodynamic model “falls apart” and can not feasibly connect the environmental redox conditions to the carbon oxidation states of both types of biomacromolecules. However, the average composition of the proteins inferred from the metagenome in the uppermost sample of the ETNP OMZ is relatively oxidized (Figure 2J), giving it a positive relative affinity, as shown by the blue dot on the bold blue line for this dataset in Figure 4C.

### 3.7. Inverse Trends in Oceans

The carbon oxidation state of metagenomic DNA increases with depth in OMZs (Figures 2I,K) and at station HOT ALOHA in the subtropical North Pacific open-ocean gyre (Figure S1). Because oxygen concentrations are actually lower at depth, we investigated the literature and datasets in greater detail to find an explanation for this inverse trend of *Z*_C_.

We consider four alternative explanations. First, horizontal gene transfer (HGT) in some environments could impact the composition of metagenomes. A possible impact of HGT on chemical composition of DNA is supported by experiments with *Salmonella* showing the silencing of low GC content sequences acquired from foreign DNA, and a preference for AT-rich sequences in “selfish” genetic elements (Navarre et al., 2006). In addition, the proportions of mobile genetic elements are higher in vent metagenomes compared to oceans (Anderson et al., 2014), and bacteria and archaea are more prone to gene sharing in high-temperature and anaerobic settings (Fuchsman et al., 2017).

A second candidate explanation could be provided by downward transport of DNA from near-surface waters on sinking particles. Previous authors have noted that downward transport of DNA adsorbed to particles could increase the GC content of metagenomes from deeper regions (Eloe et al., 2011), which would also give a higher *Z*_C_ (Figure 1B). Large particles, or the guts of eukaryotes that inhabit the particles, are hot spots for microbial activity that are likely to develop anoxic microniches (Fontanez et al., 2015). Therefore, the contribution of sinking particles to metagenomic DNA at depth is probably derived from both the ocean surface and anoxic microniches in the particles. Although particle transport could provide for some surface-derived features at depth, at best it would tend to flatten the compositional patterns and can not explain the strong inverse trends we see for the OMZs in Figure 2.

**Figure 5:**
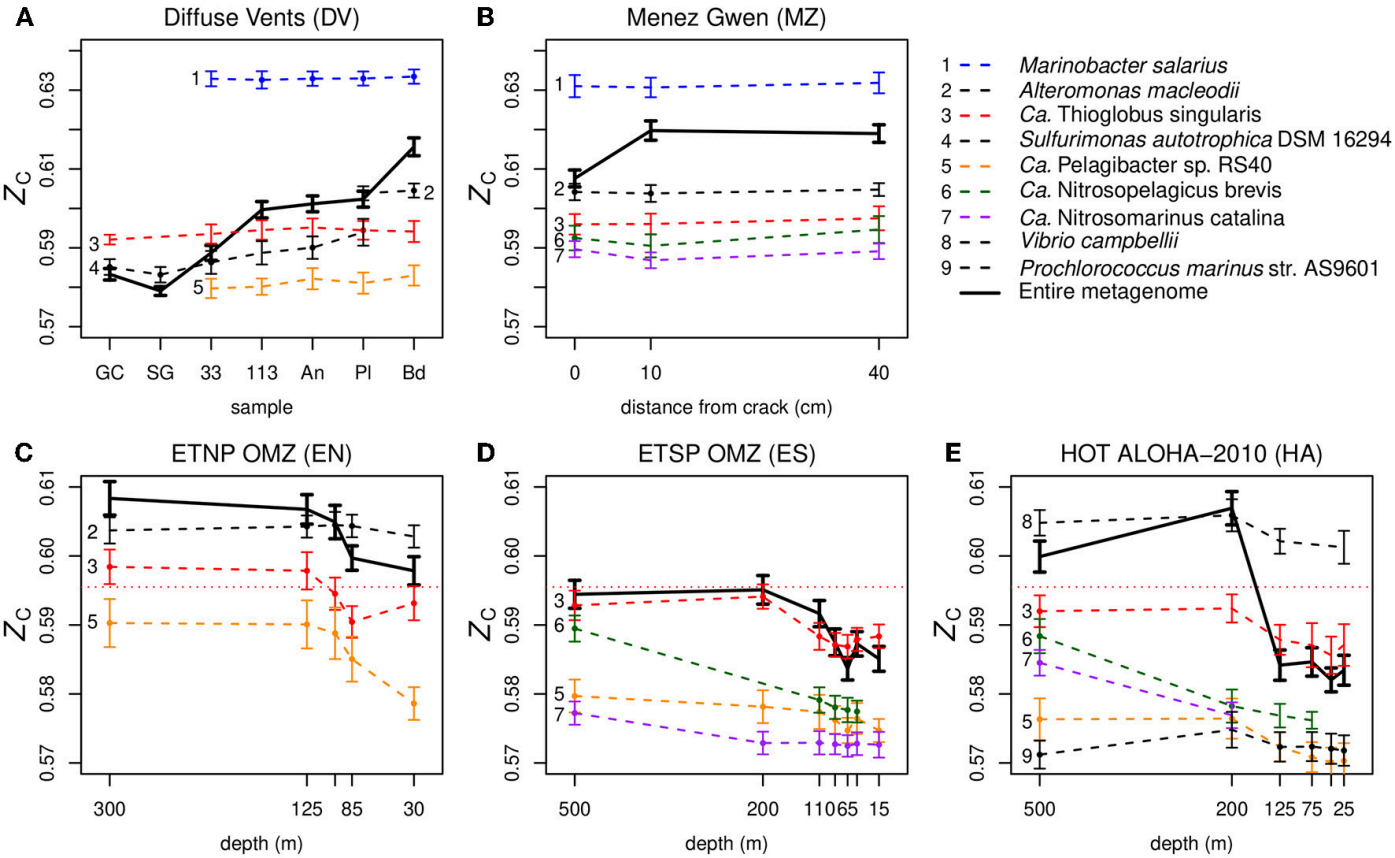
Carbon oxidation state of DNA sequences for individual species in metagenomes from different types of marine environments: **(A,B)** hydrothermal vents, **(C,D)** oxygen minimum zones, and **(E)** relatively oxic ocean gyre. Colors are used to identify species that are present in multiple datasets, small dots indicate species with reads that make up >1% of the total number of classified reads in that sample, and bold lines indicate the entire metagenomes. The horizontal dotted line in the lower plots indicates the average *Z*_C_ of DNA from *Ca*. Thioglobus singularis in the vent datasets. The calculations for HOT ALOHA shown here use a more recent and larger dataset than the one shown in Figure S1; see Appendix for description. To avoid clutter from the high density of near-surface samples, depths greater than 500 m at ETSP OMZ and HOT ALOHA are not shown here. Results for the deeper samples are shown in Figure S3. The bold lines in these plots represent entire metagenomes (after cleaning and dereplication), which are larger than the partial datasets used to make Figure 2 and Figures S1, S2; the only significant difference is the higher *Z*_C_ apparent here for the 10 cm Menez Gwen sample.

A third candidate explanation comes from a recent paper by Mende et al. (2017), who identified increasing GC content in metagenomes below the mesopelagic zone at HOT ALOHA. Parallel patterns in GC content were observed for many clades, and could be attributed to selection for low GC content associated with genome reduction in nitrogen-limited surface waters (Grzymski and Dussaq, 2012). Nitrogen limitation impacts the usage patterns of both codons and amino acids; not only does the GC base pair have one more nitrogen atom than the AT base pair, but the amino acids coded by nitrogen-rich codons also have more nitrogen (Bragg and Hyder, 2004). The observation by Mende et al. (2017) of increasing GC content with depth corroborates our findings of higher carbon oxidation state of metagenomic DNA in the deeper water at HOT ALOHA (Figures 1B, 5E; Figure S1).

The fourth candidate explanation is connected with our observation of variable *Z*_C_ at the level of species. We obtained taxonomic classifications using Kraken (Wood and Salzberg, 2014) (see section Methods) and calculated the species-level carbon oxidation state for selected relatively abundant species (Figure 5). The absence of representative species with *Z*_C_ higher than the whole-metagenome average in Figure 5 is probably due to limitations of the reference database used for taxonomic classification or the occurrence of relatively numerous but low-abundance species with high genomic *Z*_C_ (i.e., high GC content). As shown in Figures 5A,B, DNA sequences for most of the species identified have only slightly variable *Z*_C_ along redox gradients in hydrothermal vents (Diffuse Vents and Menez Gwen), which is the expected outcome if the metagenomes are random samples of the intracellular DNA from different species, each with a constant genome composition. On the other hand, metagenomic sequences of some species in the oxygen-minimum zones and HOT ALOHA exhibit a systematic variation in *Z*_C_ that is parallel to the changes in the entire metagenome instead of relatively constant (Figures 5C–E). This is particularly evident for sequences assigned to *Ca*. Thioglobus singularis, which has nearly constant *Z*_C_ in the vents, but a variable *Z*_C_ that tracks the differences at the metagenomic level in the OMZ datasets.

Keeping in mind that the species-level assignments used here should be interpreted with care (see section Methods for details), variability of the carbon oxidation state of DNA in oceanic metagenomes is visible even at the species level (Figures 5C–E). We suggest that pervasive metagenomic reshaping, which might affect taxonomic levels even lower than the genus- to phylum-level transitions reported by Mende et al. (2017), might be an indicator of some extracellular process that modifies metagenomic DNA. A recent study reported the preferential removal of low-GC extracellular DNA due to heterotrophic degradation in an anoxic sediment (Vuillemin et al., 2017), which may be due in part to faster bacterial degradation of adenosine monophosphate than cytidine monophosphate (Therkildsen et al., 1996; Dell'Anno et al., 2002). Heterotrophic microbes are abundant in OMZs, where they are responsible for the depletion of oxygen (Stewart et al., 2012). If they selectively degrade extracellular DNA that is rich in A–T base pairs (which is relatively reduced; see Figures 1A,B), it could potentially bias the entire metagenomic DNA pool to higher GC content and account for the changes in *Z*_C_ at the species level in the OMZs and HOT ALOHA (Figures 5C–E). However, this explanation depends on a significant representation of extracellular DNA in the metagenomes. Dissolved extracellular DNA is plentiful in seawater (Nagler et al., 2018), and considering the tendency for fine particles to adsorb DNA (Liang and Keeley, 2013), extracellular DNA that passes through a prefilter might be encountered in the filtrate used for metagenomic analysis. In addition, some dissolved DNA can be adsorbed to filter material, although its contribution to metagenomes becomes proportionally smaller with higher filtering volumes (Boström et al., 2004). Besides these potential direct contributions of extracellular DNA to metagenomes, we speculate that extracellular processing of mobile genetic elements could modify the composition of foreign DNA that is subjected to HGT, which would then be incorporated as cellular genomic material that is detected by metagenomic analyses.

Overall, it seems that the inverse trend of *Z*_C_ in the oceans primarily reflects the transition of GC content that is driven by nutrient limitation near the surface (Mende et al., 2017). Not only is lower GC content associated with lower *Z*_C_ (Figure 1B), but selection for lower nitrogen content in proteins would also tend to decrease *Z*_C_ (Equation 1) and potentially counteract the contribution made by higher oxygen concentrations. Selective degradation of extracellular low-*Z*_C_ (AT-rich) DNA is an additional hypothesis that may explain the species-level trends, but requires confirmation of the extent of extracellular DNA represented in metagenomic data. The potential for heterotrophic degradation is not limited to the OMZs, and can occur near vents such as Menez Gwen (Meier et al., 2016). However, except for *Sulfurimonas autotrophica* in the Diffuse Vents, our data do not show significant species-level changes in the *Z*_C_ of metagenomic DNA among different vent samples (Figures 5A,B).

It appears that the geobiochemical hypothesis that redox gradients result in thermodynamic constraints on the chemical compositions of multiple types of biomacromolecules is more relevant to environments characterized by mixing of fluids or the upper surface layers of stratified systems, than to the interiors of stratified systems like oxygen minimum zones. In this sense, microbial mats like Guerrero Negro may be similar to the stratified zones of oceans. Although there is not sufficient sequence data to quantify the species-level carbon oxidation state of metagenomic DNA in the Guerrero Negro microbial mat, selective degradation of AT-rich, relatively reduced extracellular DNA might contribute to the rise of *Z*_C_ in the deeper layers of the mat (Figure 2S). Supporting this idea, ferredoxins and genes for sugar degradation pathways are more abundant in the deeper layers, indicating the genetic potential for anaerobic respiration and heterotrophic metabolism of sugars (Kunin et al., 2008).

### 3.8. Carbon Oxidation State Reflects Complex Processes in Sediments

The carbon oxidation state of DNA in the Baltic Sea sediment metagenomes and metatranscriptomes decreases between the surface and 41–42 mbsf (Figure 2A, Figure S1), tracking the transition to more reducing conditions at depth. Microbial nitrate and sulfate reduction take place in the surface sediments of the Baltic Sea, but common electron acceptors are depleted in the deeper subsurface sediments, where methanogenesis and reductive dehalogenation are likely metabolic strategies (Zinke et al., 2017). However, the deepest sampled sediments (47 m below seafloor (mbsf) at Landsort Deep and 67 and 81 mbsf at Little Belt) may also develop relatively oxidizing conditions. These deep sediments were deposited in the freshwater glacial Baltic Ice Lake (Marshall et al., 2018) and have lower organic carbon and higher iron oxide content than the overlying brackish-water sediments (Egger et al., 2017). These environmental differences may contribute to the sharp rise of *Z*_C_ of metagenomic DNA in the deeper sediments.

The depth profile of carbon oxidation state of metagenomes in sediments offshore from the Shimokita Peninsula has a V-shape with a minimum *Z*_C_ at 5.1 mbsf (Figure 2C), which coincides with the sulfate-methane transition zone (SMTZ) (Nunoura et al., 2016). Highly reducing conditions at this depth are suggested by shipboard detection of a sulfidic odor and methane in the headspace of core fluids, but not at deeper intervals (to 107 mbsf) where metagenomes were obtained (Aoike, 2007). This pattern is reversed for the culturability of aerobic heterotrophs, which minimizes at 4.8–8.0 mbsf and is higher at both 0.5 mbsf and greater depths (Kobayashi et al., 2008). The deep sediments also yield an unexpectedly high activity of catalase, an enzyme used by aerobic organisms (Kobayashi et al., 2008). This may be indirect evidence for less strongly reducing conditions below the SMTZ, which would provide an environmental context for the observed rise of metagenomic *Z*_C_. A reversal of redox zonation may be common in marine sediments, as aerobic metabolism can be fueled by upward diffusion of sulfate from ancient brines or oxygen and nitrate from underlying basaltic aquifers (D'Hondt et al., 2004). Selective degradation of extracellular DNA also offers a potential explanation for the gradual rise of *Z*_C_ of metagenomes below 5.1 mbsf offshore Shimokita Peninsula (Figure 2C), as aerobic heterotrophic microbes are present in the deep sediments (Kobayashi et al., 2008). However, for the Baltic Sea sediment (Figure 2A), the change in carbon oxidation state of metagenomic DNA is sharper and more likely associated with a paleoenvironmental transition.

A potential source of uncertainty in some of the datasets is the use of whole genome amplification, which was performed for all but the shallowest sample at Shimokita Peninsula (Kawai et al., 2014), and for the TLE sample of the Serpentinite Springs study (Brazelton et al., 2012). More work is needed to determine to what extent the overall oxidation state of metagenomic DNA and predicted proteins is altered by whole genome amplification and other sample preparation techniques.

### 3.9. Global Differences in Oxidation State of DNA and Proteins

Among all the datasets, the TLE sample from the Serpentinite Springs study has the most oxidized proteins inferred from metagenomes. This sample consists of spring fluid that mixed with a significant fraction of surface runoff from snowmelt (Brazelton et al., 2012); the other samples in that study, which were primarily rock-derived fluids, have markedly lower *Z*_C_ of both DNA and proteins (Figure 3). Other datasets with relatively oxidized proteins are the most vent-distal (plume-like) fluid of Menez Gwen, the surface of Organic Lake in Antarctica, and the Guerrero Negro microbial mat. The most reduced proteins are found in hot springs in Yellowstone, followed by the hydrothermal fluids of the Shrimp Gulley #2 and Ginger Castle vent sites at the Mid-Cayman Rise, which are part of the dataset for the Diffuse Vents in this study. Thus, in addition to the strong local correlations that are apparent in many individual datasets, there is a global trend for proteins in hydrothermal fluids to be more reduced than those in other environments. However, the oxygenated ocean water at HOT ALOHA also hosts relatively reduced DNA and proteins, while the oxygen minimum zones have more oxidized proteins, so the links between redox conditions and the carbon oxidation state at the global scale in oceans are more ambiguous.

The distribution of points in Figure 3A suggests that the carbon oxidation state of DNA, in contrast to proteins, falls into two groups, with relatively low and high *Z*_C_ in marine and terrestrial environments, respectively. Note that although Organic Lake is near the shore of Antarctica and has a marine-derived biota, it experiences input from terrestrial sources such as penguins and algae (Yau et al., 2013), and is classified as a terrestrial environment here. Metagenomic DNA from deep sediments of the Baltic Sea has a relatively high *Z*_C_, but this signal can be argued to have a terrestrial origin, as these sediments were deposited in a freshwater setting (Marshall et al., 2018). The highest range of *Z*_C_ of metatranscriptomic cDNA is found for the terrestrial environment represented by Mono Lake (Figure 3B). Because *Z*_C_ of double-stranded DNA scales linearly with GC content (see Figure 3B), our findings are consistent with previous reports of significantly higher whole-genome GC content in terrestrial organisms than marine organisms (Wu et al., 2012).

Multiple environmental factors impact the GC content, and fully sequenced genomes of aerobic organisms also exhibit higher GC content than those of anaerobic organisms (Naya et al., 2002). Several biological explanations can be invoked for this trend, including different patterns of amino acid utilization, higher stability of the G–C base pair, and greater codon degeneracy (Naya et al., 2002). The thermodynamic analysis presented above implies that relatively oxidizing environments favor the usage of the G–C pair owing to its higher carbon oxidation state compared to A–T (see Figure 1A), so we suggest that environmental shaping of chemical composition is another factor that contributes to the higher GC content in aerobic organisms.

### 3.10. Prospects for a Paleoredox Indicator

There is little doubt that microbial community composition dictates to a large extent the chemical composition of metagenomic DNA. However, there are many different combinations of microbial assemblages that are identified in datasets where carbon oxidation state is correlated with the redox gradient (Table 1). We therefore suggest that the major trends in carbon oxidation state emerge mainly from environmental rather than phylogenetic constraints.

**Table 1:** Major taxonomic groups in datasets where carbon oxidation states of DNA and proteins are positively correlated with the geochemical redox gradient.

**Location and References**	**Reducing**	**Transition**	**Oxidizing**
Baltic Sea Sediment (Thureborn et al., 2016; Zinke et al., 2017)	Euryarchaeota, Atribacteria, Chloroflexi	Atribacteria, Euryarchaeota, Chloroflexi, Deltaproteobacteria	Cyanobacteria, Euryarchaeota, Deltaproteobacteria
Bison Pool (Dick and Shock, 2013)	Aquificae, Crenarchaeota	Deinococcus-Thermus, Firmicutes	Chloroflexi, Cyanobacteria
Diffuse Vents (Reveillaud et al., 2016; Fortunato et al., 2018)	Archaeoglobaceae, Epsilonproteobacteria	Epsilonproteobacteria, Gammaproteobacteria	Alphaproteobacteria, Gammaproteobacteria, Nitrosopumilus
Menez Gwen (Meier et al., 2016)	Epsilonproteobacteria, Aquificae	Gammaproteobacteria, Alphaproteobacteria (Rhodobacterales)	Gammaproteobacteria, Alphaproteobacteria (SAR11)
Mono Lake (Edwardson and Hollibaugh, 2017)	Firmicutes, Proteobacteria (Deltaproteobacteria, Clostridia)	Firmicutes, Proteobacteria (Gammaproteobacteria)	Bacteriodetes, Actinobacteria
SYNH Mud Volcano (Cheng et al., 2012)	Methanomicrobiales, Methanosarcinales, Deltaproteobacteria, Bacteroidetes	Methanomicrobiales, Methanosarcinales, Firmicutes, Bacteroidetes	Methanosarcinales, ANME-1, Cyanobacteria, Gammaproteobacteria
Serpentinite Springs (Brazelton et al., 2012)	Thiomicrospira	Burkholderiales (dominant), Firmicutes	Burkholderiales, Firmicutes

*Taxonomic summaries are taken from the cited references*.

Environmental constraints on carbon oxidation state could play a key role in microbial community assembly. Foerstner et al. (2005) observed the slow timescale of genomic evolution compared to community dynamics and argued that “community GC-content patterns originate at the time of community assembly, by selective pressures restricting the set of appropriate organisms from a larger pool of available organisms.” Because it is strongly related to GC content, patterns in *Z*_C_ may have a similar origin. Our results suggest that redox conditions provide an important selective pressure, since differences in *Z*_C_, of nucleic acids as well as proteins, are the predicted consequence of thermodynamic forces acting within a redox gradient.

Projecting events in geological history onto phylogenetic trees is an attractive goal for paleoenvironmental studies (Shock and Boyd, 2015), but accurate representations require time calibration of evolutionary steps as well as development and verification of sequence proxies for environmental conditions (Boussau and Gouy, 2012). A recent study found that the carbon oxidation state of proteomes from genomes of organisms bearing different isoforms of the nitrogenase gene (Nif-A, Nif-B, Nif-C, and Nif-D) is linked to the evolutionary transition from anaerobic to aerobic metabolism (Poudel et al., 2018). Additional research aimed at clarifying the evolutionary trajectory of *Z*_C_ and other dimensions of biomolecular composition could uncover deeper links with Earth's changing environments.

Here, we suggest a conservative outlook for using the carbon oxidation state of DNA as a novel paleoredox proxy. Within marine environments, the compositional trends must be interpreted carefully, as negative correlations between carbon oxidation state and environmental O_2_ concentrations are likely. However, the Baltic Sea sediment dataset reveals a strong link between an oxidizing paleoenvironment (less organic carbon and more iron oxides) and higher *Z*_C_ of DNA sequences, but not proteins (Figures 2A,B). Although the metagenomic DNA from these sediments is derived mainly from modern organisms, its carbon oxidation state reflects changes in mineralogy and geochemical conditions brought on by a geological process.

As a more global prediction for paleoredox applications, we would expect a shift from reducing hydrothermal fluids to oxidizing marine and freshwater environments to be reflected more strongly in the carbon oxidation state of ancient proteins than DNA (see Figure 3). Although we have focused on community-wide metagenomic trends in this study, systematic differences in carbon oxidation state of proteins also occur within phylogenetic lineages, which is evident for some phyla that inhabit both high- and low-temperature (i.e., reducing and oxidizing) locations in Bison Pool (Dick and Shock, 2013). Groups that display ambiguous and smaller changes in carbon oxidation state, such as the Proteobacteria (see Figure 1 in Dick and Shock, 2013), may be less attractive for potential paleoredox applications. Taken together, these results indicate new opportunities for developing a biomacromolecular paleoredox proxy in sediment environments and for identifying ancient systems dominated by hydrothermal input, but the extension to ocean environments is more challenging.

## 4. Conclusions

We have shown that the oxidation state of carbon in DNA and protein sequences derived from metagenomes changes systematically along geochemical redox gradients. A geobiochemical hypothesis for the positive correlations is that redox gradients result in thermodynamic constraints on the chemical compositions of different types of biomacromolecules. We derived support for this hypothesis from a thermodynamic model that accounts for the overall positive correlations of oxidation states of DNA and proteins with redox gradients in hot springs and submarine hydrothermal systems.

This systematic behavior is reversed in oceanic oxygen minimum zones, yielding strong negative correlations between biomolecular oxidation state and oxygen concentration with depth. We recognize that a thermodynamic model is not applicable in these cases. It might be that the geobiochemical hypothesis is more applicable to redox gradients associated with mixing of fluids than stratified systems. In the latter, biological processes including genome reduction and horizontal gene transfer, possibly influenced by selective degradation of extracellular DNA, probably dominate. However, positive correlations are evident in particular layered systems, such as hypersaline lakes and the uppermost layers of oceans and a microbial mat. More work is needed to identify the evolutionary and ecological factors that allow the compositions of biomacromolecules in these environments to be shaped by the redox gradients.

This study promotes a perspective in which life emerges from, and is part of, the environment. Just as evolutionary constraints are regarded as limitations on the variability available to natural selection (Schwenk, 1995), the manifestation of putative thermodynamic constraints is not an indicator of biological adaptation to geochemical gradients, but of limitations on the chemical compositions of biomolecules. Our exploration of these constraints is a novel counterpart to functional studies in geomicrobiology and is a source of independent predictions linking biological and geochemical data. The carbon oxidation state can be calculated for annotated genes, hypothetical genes, and non-coding sequences, making it applicable to a wider range of sequence data than is available for taxonomic and functional analysis. Further quantifying this variable and characterizing the thermodynamic constraints on it may lead to new applications for geobiochemistry, such as using reconstructed ancestral sequences as a paleoredox proxy.

## Data Availability Statement

The datasets analyzed for this study can be found in the public sequence databases mentioned in the Methods, under the accession numbers given in Table S1. The datasets generated for this study can be found in the Zenodo repository (Dick et al., 2018), including the code used to analyze the sequence data, intermediate data files (sampled nucleobase and amino acid compositions), and code used to make the figures.

## Author Contributions

JD and AL conceived the study. JD wrote the code. JD, JT, and MY analyzed the data. All authors participated in writing and revising the manuscript.

### Conflict of Interest Statement

The authors declare that the research was conducted in the absence of any commercial or financial relationships that could be construed as a potential conflict of interest.

## Footnotes

^1^https://github.com/MG-RAST/pipeline accessed on 2018-03-07

^2^https://ccb.jhu.edu/software/kraken/ accessed on 2018-05-27

^3^https://trace.ncbi.nlm.nih.gov/Traces/sra/sra.cgi?view=software accessed on 2018-05-28

^4^http://www.kazusa.or.jp/codon/ accessed on 2018-03-21
