# Supplementary material for: Changes in Carbon Oxidation State of Metagenomes Along Geochemical Redox Gradients
Source: Front Microbiol. 2019 Feb 11;10:120. doi: 10.3389/fmicb.2019.00120 (PMC6378307; doi:10.3389/fmicb.2019.00120)
Supplement: Supplementary file 2 [file Data_Sheet_1.PDF]

Supplementary Material:
Changes in Carbon Oxidation State of Metagenomes along Geochemical Redox Gradients

SUPPLEMENTARY FIGURES

Figure S1. Carbon oxidation state (Z_C) of double-stranded DNA (red symbols) and messenger RNA of predicted coding sequences (blue symbols) along geochemical redox gradients. In order to plot both DNA and RNA on the same diagram, a constant of 0.28 was subtracted from Z_C of RNA. The horizontal axis in each plot is ordered so that relatively oxidizing conditions are toward the right-hand side. Abbreviations: MG – metagenome; MT – metatranscriptome; abbreviations for sample names are given in the Appendix.

Figure S2. Carbon oxidation state (Z_C) of proteins along geochemical redox gradients. Plot arrangement, labels, and abbreviations are the same as in Figure S1.

Figure S3. Carbon oxidation state of DNA sequences for individual species in metagenomes from different types of marine environments. This figure shows the same data as Figure 5 in the main text, but is extended to include the deepest samples of the ETSP OMZ and HOT ALOHA datasets.

SUPPLEMENTARY TABLES

Table S1. Sequence processing statistics.

Table S2. Taxonomic classification statistics.

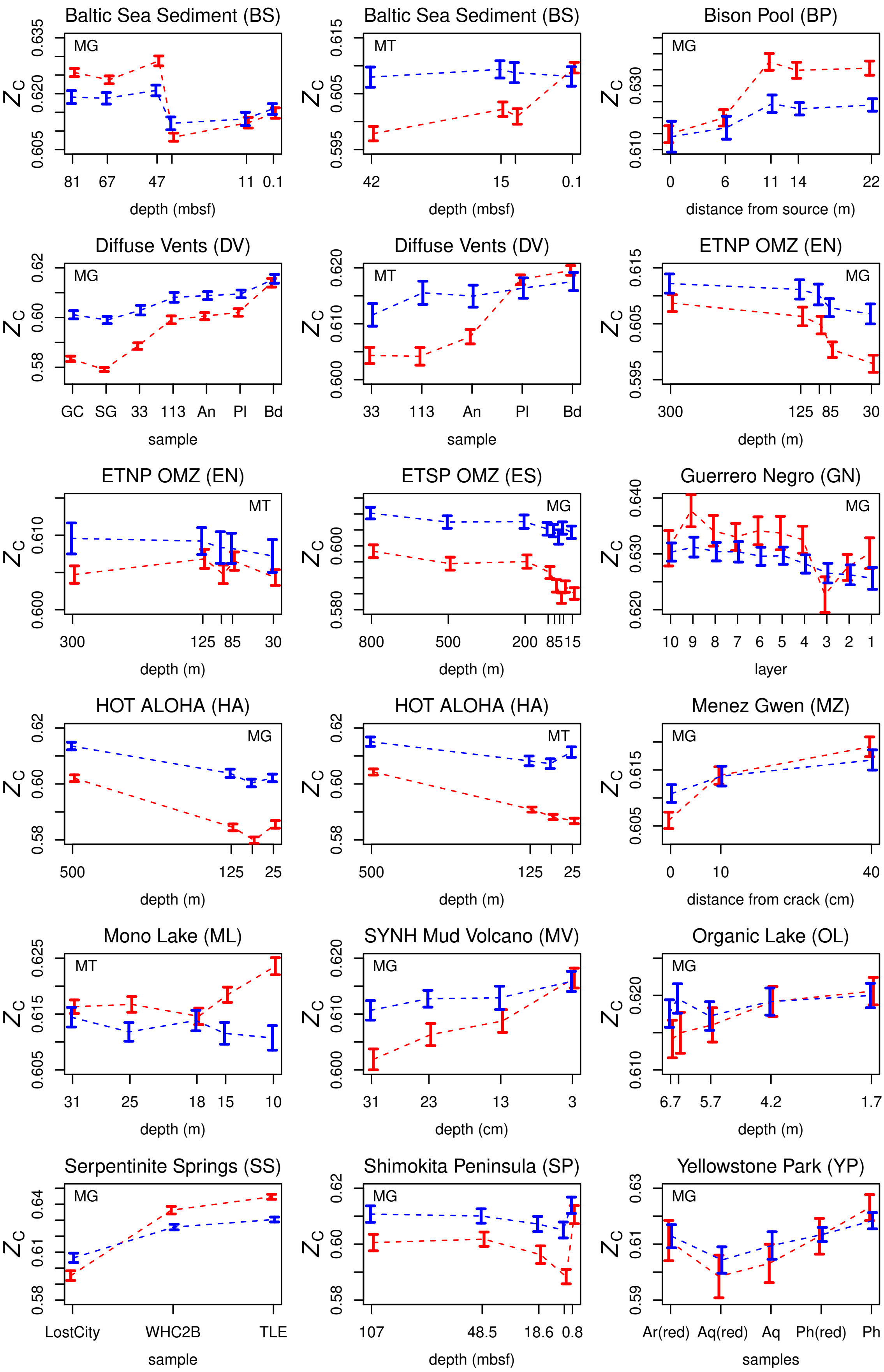
Baltic Sea Sediment (BS)
MG
ZC
0.605 0.620 0.635
81 67 47 11 0.1
depth (mbsf)
Baltic Sea Sediment (BS)
MT
ZC
0.595 0.605 0.615
42 15 0.1
depth (mbsf)
Bison Pool (BP)
MG
ZC
0.610 0.630
0 6 11 14 22
distance from source (m)
Diffuse Vents (DV)
MG
ZC
0.58 0.60 0.62
GC SG 33 113 An Pl Bd
sample
Diffuse Vents (DV)
MT
ZC
0.600 0.610 0.620
33 113 An Pl Bd
sample
ETNP OMZ (EN)
MG
ZC
0.595 0.605 0.615
300 125 85 30
depth (m)
ETNP OMZ (EN)
MT
ZC
0.600 0.610
300 125 85 30
depth (m)
ETSP OMZ (ES)
MG
ZC
0.580 0.600
800 500 200 8515
depth (m)
Guerrero Negro (GN)
MG
ZC
0.620 0.630 0.640
10 9 8 7 6 5 4 3 2 1
layer
HOT ALOHA (HA)
MG
ZC
0.58 0.60 0.62
500 125 25
depth (m)
HOT ALOHA (HA)
MT
ZC
0.58 0.60 0.62
500 125 25
depth (m)
Menez Gwen (MZ)
MG
ZC
0.605 0.615
0 10 40
distance from crack (cm)
Mono Lake (ML)
MT
ZC
0.605 0.615 0.625
31 25 18 15 10
depth (m)
SYNH Mud Volcano (MV)
MG
ZC
0.600 0.610 0.620
31 23 13 3
depth (cm)
Organic Lake (OL)
MG
ZC
0.610 0.620
6.7 5.7 4.2 1.7
depth (m)
Serpentinite Springs (SS)
MG
ZC
0.58 0.61 0.64
LostCity WHC2B TLE
sample
Shimokita Peninsula (SP)
MG
ZC
0.58 0.60 0.62
107 48.5 18.6 0.8
depth (mbsf)
Yellowstone Park (YP)
MG
ZC
0.59 0.61 0.63
Ar(red) Aq(red) Aq Ph(red) Ph
samples

Figure S1.

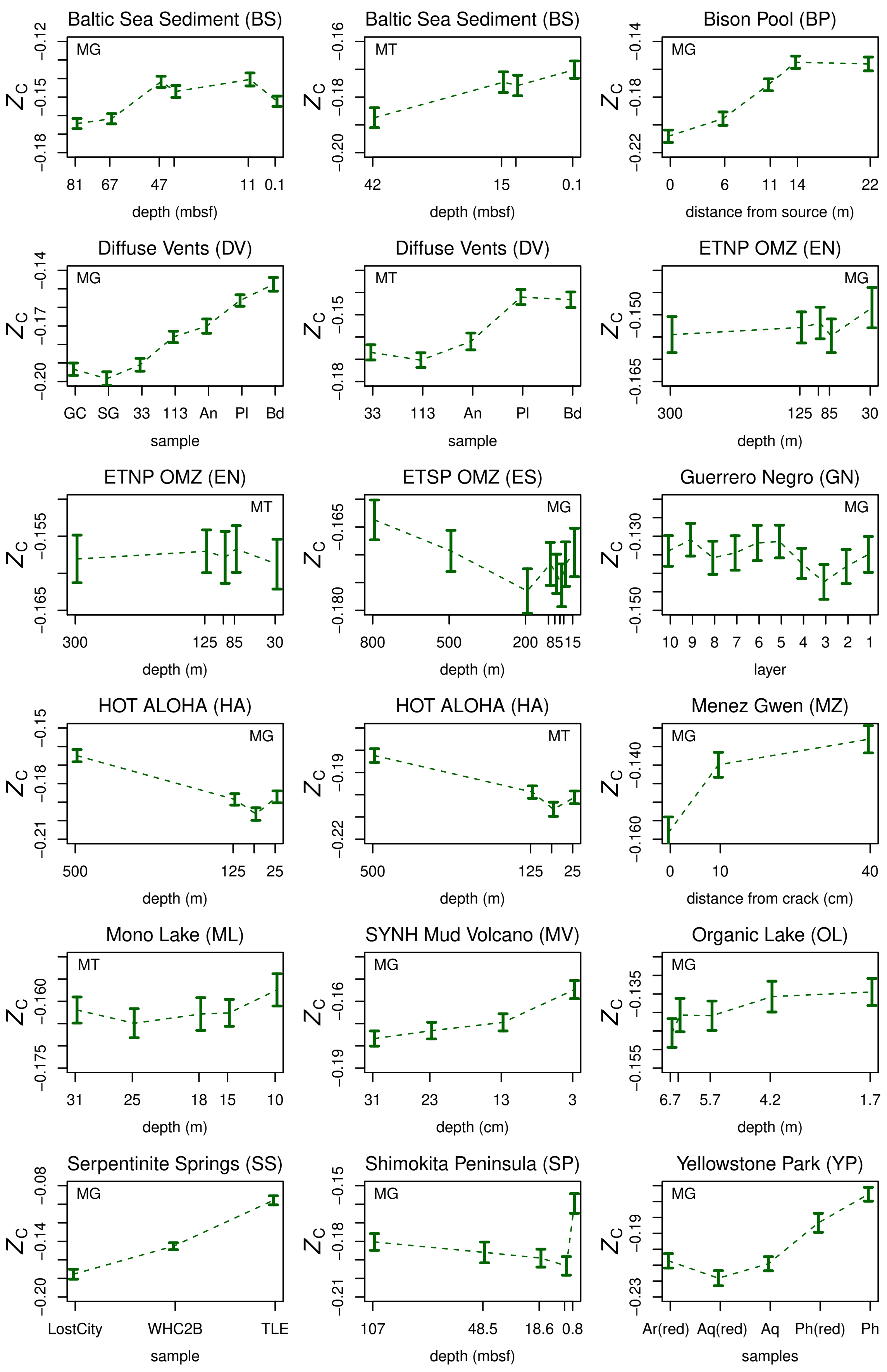
Baltic Sea Sediment (BS)
MG
Z_C
−0.18 −0.15 −0.12
81 67 47 11 0.1
depth (mbsf)
Baltic Sea Sediment (BS)
MT
Z_C
−0.20 −0.18 −0.16
42 15 0.1
depth (mbsf)
Bison Pool (BP)
MG
Z_C
−0.22 −0.18 −0.14
0 6 11 14 22
distance from source (m)
Diffuse Vents (DV)
MG
Z_C
−0.20 −0.17 −0.14
GC SG 33 113 An Pl Bd
sample
Diffuse Vents (DV)
MT
Z_C
−0.18 −0.15
33 113 An Pl Bd
sample
ETNP OMZ (EN)
MG
Z_C
−0.165 −0.150
300 125 85 30
depth (m)
ETNP OMZ (EN)
MT
Z_C
−0.165 −0.155
300 125 85 30
depth (m)
ETSP OMZ (ES)
MG
Z_C
−0.180 −0.165
800 500 200 85 15
depth (m)
Guerrero Negro (GN)
MG
Z_C
−0.150 −0.130
10 9 8 7 6 5 4 3 2 1
layer
HOT ALOHA (HA)
MG
Z_C
−0.21 −0.18 −0.15
500 125 25
depth (m)
HOT ALOHA (HA)
MT
Z_C
−0.22 −0.19
500 125 25
depth (m)
Menez Gwen (MZ)
MG
Z_C
−0.160 −0.140
0 10 40
distance from crack (cm)
Mono Lake (ML)
MT
Z_C
−0.175 −0.160
31 25 18 15 10
depth (m)
SYNH Mud Volcano (MV)
MG
Z_C
−0.19 −0.16
31 23 13 3
depth (cm)
Organic Lake (OL)
MG
Z_C
−0.155 −0.135
6.7 5.7 4.2 1.7
depth (m)
Serpentinite Springs (SS)
MG
Z_C
−0.20 −0.14 −0.08
LostCity WHC2B TLE
sample
Shimokita Peninsula (SP)
MG
Z_C
−0.21 −0.18 −0.15
107 48.5 18.6 0.8
depth (mbsf)
Yellowstone Park (YP)
MG
Z_C
−0.23 −0.19
Ar(red) Aq(red) Aq Ph(red) Ph
samples

Figure S2.

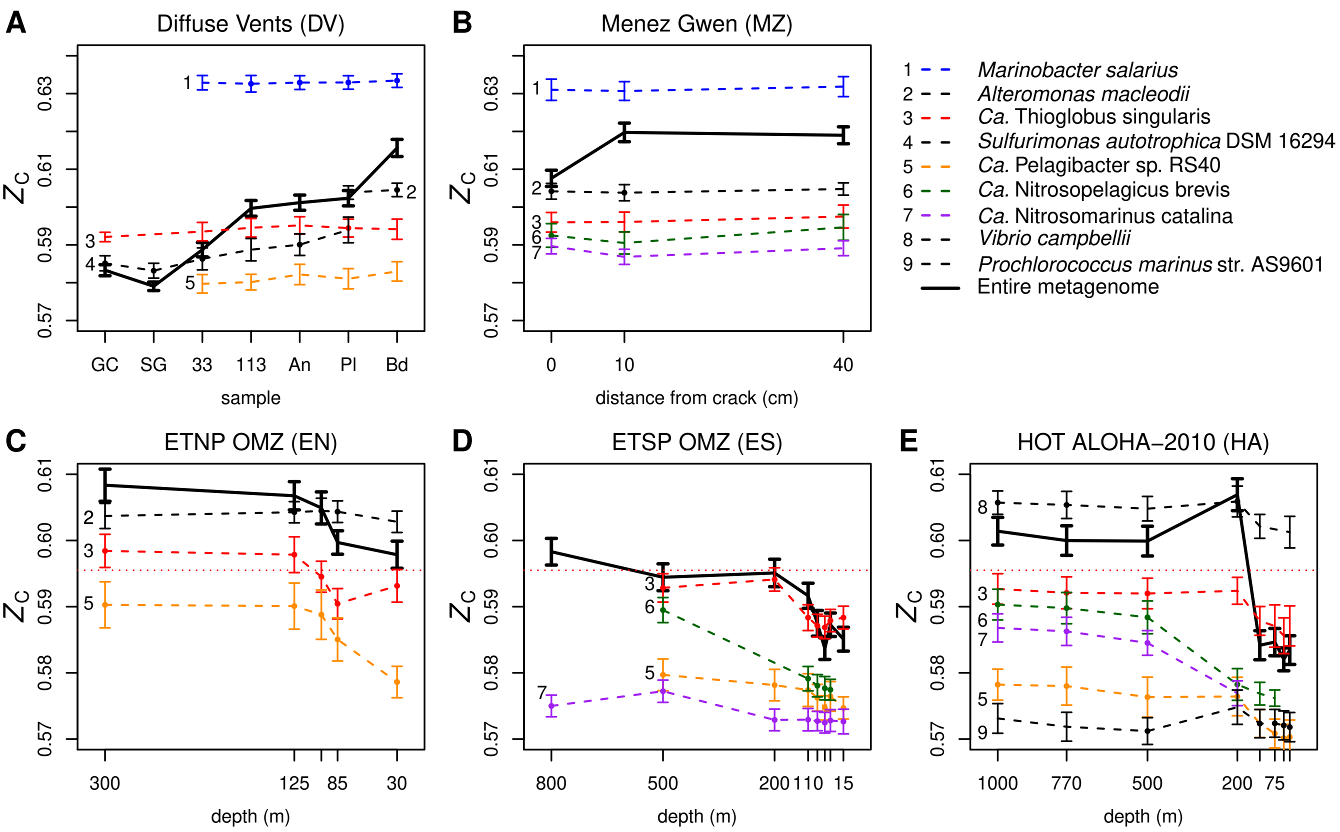

A
Diffuse Vents (DV)
B
Menez Gwen (MZ)
C
ETNP OMZ (EN)
D
ETSP OMZ (ES)
E
HOT ALOHA–2010 (HA)
Z_C
sample
GC SG 33 113 An Pl Bd
distance from crack (cm)
0 10 40
depth (m)
300 125 85 30
800 500 200 110 15
1000 770 500 200 75
0.57 0.59 0.61 0.63
0.57 0.58 0.59 0.60 0.61
1 Marinobacter salarius
2 Alteromonas macleodii
3 Ca. Thioglobus singularis
4 Sulfurimonas autotrophica DSM 16294
5 Ca. Pelagibacter sp. RS40
6 Ca. Nitrosopelagicus brevis
7 Ca. Nitrosomarinus catalina
8 Vibrio campbellii
9 Prochlorococcus marinus str. AS9601
Entire metagenome

Figure S3.
