# Supplementary material for: Changes in Carbon Oxidation State of Metagenomes Along Geochemical Redox Gradients
Source: Front Microbiol. 2019 Feb 11;10:120. doi: 10.3389/fmicb.2019.00120 (PMC6378307; doi:10.3389/fmicb.2019.00120)
Supplement: Supplementary file 3 [file Data_Sheet_2.pdf]

Appendix: Changes in Carbon Oxidation State of Metagenomes along Geochemical Redox Gradients

Each paragraph below gives references to the original publications of sequencing data and general characteristics of the field areas, including a summary of the overall redox gradient. Abbreviations for datasets are shown in parentheses, and abbreviations in square brackets indicate whether the data are for metagenomes [MG] or metatranscriptomes [MT].

Baltic Sea Sediment (BS) [MG, MT]

Thureborn et al. (2016) reported metagenomic and metatranscriptomic data for surface sediments (0–10 cm below seafloor) at Landsort Deep (466 m water depth), Zinke et al. (2017) reported metatranscriptomes for subsurface sediments 12 m below seafloor at site 63E of the Landsort Deep and 15 and 42 m below seafloor at site M59 in the Little Belt, where water from the North Sea enters the Baltic Sea, and Marshall et al. (2018) reported metagenomic data for four sites including the Landsort Deep (M63E) at 11 and 47 mbsf and Little Belt (M59E) at 41, 67, and 81 mbsf. Although the near-surface redox gradient is more oxidizing toward the surface, the conditions may also be relatively oxidizing in the deepest sediments (47 mbsf at Landsort Deep and 67 and 81 mbsf at Little Belt), which were deposited during a period of freshwater in the glacial Baltic Ice Lake (Marshall et al., 2018).

Bison Pool (BP) [MG]

Havig et al. (2011) and Swingley et al. (2012) reported metagenomes of biofilms sampled from five sites at Bison Pool, an alkaline hot spring located in the Lower Geyser Basin of Yellowstone National Park. At the time of biofilm sampling in 2005, the dissolved O_2 increased from 0.2 mg/L at 93 °C (site 1, source pool) to 0.9 mg/L in the "photosynthetic fringe" at 67 °C (site 3), then more sharply up to 2.7 mg/L at 57 °C (site 5, 22 m from the source pool) (Swingley et al., 2012). Cooler temperatures are also associated with higher sulfate/sulfide ratios and higher oxidation-reduction potential (Dick and Shock, 2011). The redox gradient is more oxidizing going away from the source pool.

Diffuse Vents (DV) [MG, MT]

Reveillaud et al. (2016) reported metagenomic data for the Ginger Castle and Shrimp Gulley #2 vent sites, hosted in ultramafic and mafic rocks, respectively, in the Von Damm and Piccard vent systems on the Mid-Cayman Rise (western Caribbean), and Fortunato et al. (2018) reported metagenomic and metatranscriptomic data for diffuse vents from Axial Seamount on the Juan de Fuca Ridge (NE Pacific Ocean). H_2 concentrations at Ginger Castle and Shrimp Gulley #2 were 11.3 and 0.41 mM (Reeves et al., 2014). H_2 concentrations measured at Juan de Fuca Ridge in 2015 were 12.5 µM (Anemone vent), 0.3 µM (Marker 113), and 0.1 µM (Marker 33); H_2 concentrations are presumably lower in the plume above the Anemone vent and below detection in background seawater. Considering the temperature fluctuations at Anemone and the higher seawater mixing ratio at Marker 113 than Marker 33 (Fortunato et al., 2018), the overall redox gradient is (more oxidizing last): Ginger Castle (GC), Shrimp Gulley #2 (SG), Marker 33 (M33), Marker 113 (M113), Anemone (Anem), Anemone plume (Pl), and seawater (Bd).

ETNP OMZ (EN) [MG, MT]

Glass et al. (2015) and Ganesh et al. (2015) reported metagenomic and metatranscriptomic data for a depth transect in the Eastern Tropical North Pacific oxygen minimum zone (ETNP OMZ). Dissolved oxygen concentrations at Station 6 were 200 µM at 30 m (upper oxic zone), 0.4 µM at 85 m (base of upper oxycline), and 25 nM to ~9 nM at 100 m, 125 m, and 300 m (including the upper and core OMZ; O_2 measurements from Figure 1 of Ganesh et al., 2015). We selected metatranscriptomic data for the free-living size fraction (0.2–1.6 µm). The overall redox gradient is more oxidizing toward the surface.

ETSP OMZ (ES) [MG]

Stewart et al. (2012) reported metagenomic and metatranscriptomic data for the Eastern Tropical South Pacific oxygen minimum zone (ETSP OMZ). Oxygen concentrations at 50 and 85 m were <1 µM and at 110 m and 200 m were in the nM range. The archived metagenomic datasets (under SRA023632) include four other depths not described in Stewart et al., 2012, but that were used in this study: 15 m, 65 m, 500 m, and 800 m. The associated metatranscriptomic data were not used in this study because they are limited to 200 m. The redox gradient is more oxidizing toward the surface.

Guerrero Negro (GN) [MG]

Kunin et al. (2008) reported metagenomes from different layers of the Guerrero Negro microbial mat (Baja California Sur, Mexico). Layers 1–2 correspond to the photic/oxic zone (0.5 and 1.5 mm average sampling depth), layers 3–6 were taken from the oxic-anoxic border (2.5, 3.5, 4.5, and 5.5 mm, low H_2S), and layers 7–10 are from the anoxic zone (8, 16, 28, and 41.5 mm, high H_2S). The redox gradient is more oxidizing toward the surface; however, the surface layers also become anoxic at night when photosynthesis stops while microbial sulfate reduction continues (Kunin et al., 2008).

HOT ALOHA (HA) [MG, MT]

Shi et al. (2011) reported metagenomic and metatranscriptomic data collected in March 2006 from four depths at the Hawaii Ocean Time-Series (HOT) station ALOHA. The oxygen concentration decreased from ca. 200 to 125 µmol/kg between the surface and 500 m (Figure S1 of Shi et al., 2011); the minimum oxygen concentration is greater than those encountered in permanent oxygen minimum zones such as ETNP and ETSP. The overall redox gradient is more oxidizing toward the surface. For taxonomic classification (**Figure 5**), we used the larger metagenomic dataset for samples collected in September 2010, which is one of a time series reported by Mende et al. (2017).

Menez Gwen (MZ) [MG]

Meier et al. (2016) reported metagenomic data for three locations in the “Woody” diffuse venting site of the Menez Gwen hydrothermal field on the Mid-Atlantic ridge. Water samples were from locations identified as WdCr-f2 (a crack in the rock where the diffuse fluid emerges), Wd-10L (10 cm to the left of the crack), and Wd-40UP (40 cm above the rock). Mixing models show elevated concentrations of H_2, CH_4, and H_2S, and slightly lower O_2, at WdCr-f2 compared to the other locations (Table 1 of Meier et al., 2016). The overall redox gradient is more oxidizing going from WdCr-f2 to Wd-10L to Wd-40UP.

Mono Lake (ML) [MT]

Edwardson and Hollibaugh (2017) reported metatranscriptomic data for five depths at a station in Mono Lake (California). Measured oxygen concentrations decreased from ca. 5 mg/l at 10 m depth to 0.83 mg/l

at 15 m and were below the detection limit (0.68 mg/l) at other depths. The 15 and 18 m depths are considered to be the redox transition zone. Sulfide concentrations were below 2 μM at these depths, but increased to 8 and ca. 20 μM at 25 and 31 m, respectively (Figure 1 of Edwardson and Hollibaugh, 2017). The redox gradient is more oxidizing toward the surface.

SYNH Mud Volcano (MV) [MG]

Cheng et al. (2012) reported metagenomic data for sediment in the mud platform adjacent to a bubbling mud pool at the Shin-Yan-Ny-Hu (SYNH) Mud Volcano in Southwestern Taiwan. Sampling depths were 3, 13, 23, and 31 cm. Oxygen concentrations were not reported, but the measured sulfate concentrations in a neighboring core decreased from 4.1 mM at the surface to $<$ 10 μM at at depths greater than 10 cm, and methane concentrations increased from ca. 0.4 mM at the surface to greater than 1.29 mM at 20 cm depth. The redox gradient is more oxidizing toward the surface.

Organic Lake (OL) [MG]

Yau et al. (2013) reported metagenomic data for a depth transect in the deepest part of Organic Lake, a hypersaline lake in the Vestfold Hills, Antarctica. We used metagenomic data for the 0.8 μm size fraction, which is dominated by bacteria; both the smaller (0.1 μm) and larger (3 μm) size fractions had greater numbers of eukaryotic sequences (Yau et al., 2013). Dissolved oxygen concentrations measured at the sampling depths for metagenomics (1.7, 4.2, 5.7, 6.5, and 6.7) were ca. 10, 8.6, 7.2, 6.1, and 6 % saturation, respectively (Figure 1 of Yau et al., 2013). The redox gradient is more oxidizing toward the surface.

Serpentinite Springs (SS) [MG]

Brazelton et al. (2012) reported metagenomic data for a submarine chimney and biofilms at the Lost City hydrothermal field (15 km from the Mid-Atlantic Ridge) and fluids from two seeps at the continental Tablelands Ophiolite (Newfoundland, Canada). The measured concentrations of H_2 at Lost City was 13 mmol/kg (Brazelton et al., 2012). The first sample at the Tablelands Ophiolite, WHC2b, was collected from the bottom of a pool with pH 12 and Eh -733 mV, with a measured H_2 concentration of 0.24 mmol/kg at the time of sampling. The second sample, TLE, was collected from a travertine spring that was mixing with surface fluids derived from snow melt and had a lower pH (10.5) and higher Eh (+25 mV), and undetectable H_2 at the time of sampling. The redox gradient is more oxidizing going from Lost City to WHC2b to TLE.

Shimokita Peninsula (SP) [MG]

Kawai et al. (2014) reported metagenomic data for a sediment core off the Shimokita Peninsula in northeast Japan at five depths (0.8, 5.1, 18.6, 48.5, and 107.0 mbsf). Near-subsurface sulfate reduction is supported by the detection of methane near the 5.1 m interval, but not at the other depths where metagenomic data were obtained (Aoike, 2007). The experimental growth of cultivable aerobic organisms minimizes at 4.8 to 8.0 mbsf and is highest at 210–220 mbsf (Kobayashi et al., 2008). The available evidence suggests a V-shaped redox gradient with most reducing conditions at 5.1 mbsf.

Yellowstone Park (YP) [MG]

Inskeep et al. (2013) reported metagenomic data for 20 sites in Yellowstone National Park including community types classified as archaeal (in sediments), Aquificales (streamer biofilms in flowing water) and phototrophic mats. According to the presence of sulfide and/or elemental S (Inskeep et al., 2013), the

archaeal communities (6 sites) are reducing, the Aquificales communities are split equally among reducing and relatively non-reducing conditions (3 sites each), and the phototrophic communities are mostly non-reducing (4 sites), with a smaller number classified as reducing (2 sites). We classified the Aquificales community at 100 Spring Plain (OSP_14) as relatively non-reducing because it has a higher oxygen concentration than the other sulfidic sites (Table S2 of Inskeep et al., 2013). Sequence data in each group were pooled for analysis in this study. The overall redox gradient is more oxidizing going from the archaeal (Ar(red)) to Aquificales (Aq(red) and Aq) to phototrophic (Ph(red) and Ph) communities.

REFERENCES

Aoike, K. (2007). *CK06-06 D/V Chikyu Shakedown Cruise Offshore Shimokita*. Laboratory operation report, JAMSTEC Center for Deep Earth Exploration, Yokosuka, Japan

Brazelton, W., Nelson, B., and Schrenk, M. (2012). Metagenomic evidence for H_2 oxidation and H_2 production by serpentinite-hosted subsurface microbial communities. *Frontiers in Microbiology* 2, 268. doi:10.3389/fmicb.2011.00268

Cheng, T.-W., Chang, Y.-H., Tang, S.-L., Tseng, C.-H., Chiang, P.-W., Chang, K.-T., et al. (2012). Metabolic stratification driven by surface and subsurface interactions in a terrestrial mud volcano. *ISME Journal* 6, 2280–2290. doi:10.1038/ismej.2012.61

Dick, J. M. and Shock, E. L. (2011). Calculation of the relative chemical stabilities of proteins as a function of temperature and redox chemistry in a hot spring. *PLoS ONE* 6, e22782. doi:10.1371/journal.pone.0022782

Edwardson, C. F. and Hollibaugh, J. T. (2017). Metatranscriptomic analysis of prokaryotic communities active in sulfur and arsenic cycling in Mono Lake, California, USA. *ISME Journal* 11, 2195–2208. doi:10.1038/ismej.2017.80

Fortunato, C. S., Larson, B., Butterfield, D. A., and Huber, J. A. (2018). Spatially distinct, temporally stable microbial populations mediate biogeochemical cycling at and below the seafloor in hydrothermal vent fluids. *Environmental Microbiology* 20, 769–784. doi:10.1111/1462-2920.14011

Ganesh, S., Bristow, L. A., Larsen, M., Sarode, N., Thamdrup, B., and Stewart, F. J. (2015). Size-fraction partitioning of community gene transcription and nitrogen metabolism in a marine oxygen minimum zone. *ISME Journal* 9, 2682. doi:10.1038/ismej.2015.44

Glass, J. B., Kretz, C. B., Ganesh, S., Ranjan, P., Seston, S. L., Buck, K. N., et al. (2015). Meta-omic signatures of microbial metal and nitrogen cycling in marine oxygen minimum zones. *Frontiers in Microbiology* 6, 998. doi:10.3389/fmicb.2015.00998

Havig, J. R., Raymond, J., Meyer-Dombard, D. R., Zolotova, N., and Shock, E. L. (2011). Merging isotopes and community genomics in a siliceous sinter-depositing hot spring. *Journal of Geophysical Research* 116, G01005. doi:10.1029/2010JG001415

Inskeep, W. P., Jay, Z. J., Tringe, S. G., Herrgard, M., and Rusch, D. B. (2013). The YNP metagenome project: Environmental parameters responsible for microbial distribution in the Yellowstone geothermal ecosystem. *Frontiers in Microbiology* 4, 67. doi:10.3389/fmicb.2013.00067

Kawai, M., Futagami, T., Toyoda, A., Takaki, Y., Nishi, S., Hori, S., et al. (2014). High frequency of phylogenetically diverse reductive dehalogenase-homologous genes in deep subseafloor sedimentary metagenomes. *Frontiers in Microbiology* 5, 80. doi:10.3389/fmicb.2014.00080

Kobayashi, T., Koide, O., Mori, K., Shimamura, S., Matsuura, T., Miura, T., et al. (2008). Phylogenetic and enzymatic diversity of deep subseafloor aerobic microorganisms in organics- and methane-rich sediments off Shimokita Peninsula. *Extremophiles* 12, 519–527. doi:10.1007/s00792-008-0157-7

Kunin, V., Raes, J., Harris, J. K., Spear, J. R., Walker, J. J., Ivanova, N., et al. (2008). Millimeter-scale genetic gradients and community-level molecular convergence in a hypersaline microbial mat. *Molecular Systems Biology* 4, 198. doi:10.1038/msb.2008.35

Marshall, I. P., Karst, S. M., Nielsen, P. H., and Jørgensen, B. B. (2018). Metagenomes from deep Baltic Sea sediments reveal how past and present environmental conditions determine microbial community composition. *Marine Genomics* 37, 58–68. doi:10.1016/j.margen.2017.08.004

Meier, D. V., Bach, W., Girguis, P. R., Gruber-Vodicka, H. R., Reeves, E. P., Richter, M., et al. (2016). Heterotrophic *Proteobacteria* in the vicinity of diffuse hydrothermal venting. *Environmental Microbiology* 18, 4348–4368. doi:10.1111/1462-2920.13304

Mende, D. R., Bryant, J. A., Aylward, F. O., Eppley, J. M., Nielsen, T., Karl, D. M., et al. (2017). Environmental drivers of a microbial genomic transition zone in the ocean's interior. *Nature Microbiology* 2, 1367–1373. doi:10.1038/s41564-017-0008-3

Reeves, E. P., McDermott, J. M., and Seewald, J. S. (2014). The origin of methanethiol in midocean ridge hydrothermal fluids. *Proceedings of the National Academy of Sciences of the United States of America* 111, 5474–5479. doi:10.1073/pnas.1400643111

Reveillaud, J., Reddington, E., McDermott, J., Algar, C., Meyer, J. L., Sylva, S., et al. (2016). Subseafloor microbial communities in hydrogen-rich vent fluids from hydrothermal systems along the Mid-Cayman Rise. *Environmental Microbiology* 18, 1970–1987. doi:10.1111/1462-2920.13173

Shi, Y., Tyson, G. W., Eppley, J. M., and DeLong, E. F. (2011). Integrated metatranscriptomic and metagenomic analyses of stratified microbial assemblages in the open ocean. *ISME Journal* 5, 999–1013. doi:10.1038/ismej.2010.189

Stewart, F. J., Ulloa, O., and DeLong, E. F. (2012). Microbial metatranscriptomics in a permanent marine oxygen minimum zone. *Environmental Microbiology* 14, 23–40. doi:10.1111/j.1462-2920.2010.02400.x

Swingley, W. D., Meyer-Dombard, D. R., Shock, E. L., Alsop, E. B., Falenski, H. D., Havig, J. R., et al. (2012). Coordinating environmental genomics and geochemistry reveals metabolic transitions in a hot spring ecosystem. *PLoS ONE* 7, e38108. doi:10.1371/journal.pone.0038108

Thureborn, P., Franzetti, A., Lundin, D., and Sjöling, S. (2016). Reconstructing ecosystem functions of the active microbial community of the Baltic Sea oxygen depleted sediments. *PeerJ* 4, e1593. doi:10.7717/peerj.1593

Yau, S., Lauro, F. M., Williams, T. J., DeMaere, M. Z., Brown, M. V., Rich, J., et al. (2013). Metagenomic insights into strategies of carbon conservation and unusual sulfur biogeochemistry in a hypersaline Antarctic lake. *ISME Journal* 7, 1944. doi:10.1038/ismej.2013.69

Zinke, L. A., Mullis, M. M., Bird, J. T., Marshall, I. P. G., Jørgensen, B. B., Lloyd, K. G., et al. (2017). Thriving or surviving? Evaluating active microbial guilds in Baltic Sea sediment. *Environmental Microbiology Reports* 9, 528–536. doi:10.1111/1758-2229.12578
